# MicroRNA profiling of human primary macrophages exposed to dengue virus identifies miRNA-3614-5p as antiviral and regulator of ADAR1 expression

**DOI:** 10.1371/journal.pntd.0005981

**Published:** 2017-10-18

**Authors:** Mayra Diosa-Toro, Liliana Echavarría-Consuegra, Jacky Flipse, Geysson Javier Fernández, Joost Kluiver, Anke van den Berg, Silvio Urcuqui-Inchima, Jolanda M. Smit

**Affiliations:** 1 Department of Medical Microbiology, University of Groningen, University Medical Center Groningen, Groningen, the Netherlands; 2 Grupo Inmunovirología, Facultad de Medicina, Universidad de Antioquia UdeA, Medellín, Colombia; 3 Department of Pathology and Medical Biology, University of Groningen, University Medical Center Groningen, Groningen, the Netherlands; Colorado State University, UNITED STATES

## Abstract

**Background:**

Due to the high burden of dengue disease worldwide, a better understanding of the interactions between dengue virus (DENV) and its human host cells is of the utmost importance. Although microRNAs modulate the outcome of several viral infections, their contribution to DENV replication is poorly understood.

**Methods and principal findings:**

We investigated the microRNA expression profile of primary human macrophages challenged with DENV and deciphered the contribution of microRNAs to infection. To this end, human primary macrophages were challenged with GFP-expressing DENV and sorted to differentiate between truly infected cells (DENV-positive) and DENV-exposed but non-infected cells (DENV-negative cells). The miRNAome was determined by small RNA-Seq analysis and the effect of differentially expressed microRNAs on DENV yield was examined. Five microRNAs were differentially expressed in human macrophages challenged with DENV. Of these, miR-3614-5p was found upregulated in DENV-negative cells and its overexpression reduced DENV infectivity. The cellular targets of miR-3614-5p were identified by liquid chromatography/mass spectrometry and western blot. Adenosine deaminase acting on RNA 1 (ADAR1) was identified as one of the targets of miR-3614-5p and was shown to promote DENV infectivity at early time points post-infection.

**Conclusion/Significance:**

Overall, miRNAs appear to play a limited role in DENV replication in primary human macrophages. The miRNAs that were found upregulated in DENV-infected cells did not control the production of infectious virus particles. On the other hand, miR-3614-5p, which was upregulated in DENV-negative macrophages, reduced DENV infectivity and regulated ADAR1 expression, a protein that facilitates viral replication.

## Author summary

Dengue is the most common mosquito-borne disease worldwide and it is an increasing global concern for public health as its etiological agent, dengue virus (DENV), keeps spreading around the globe. Currently there are no specific antiviral therapies available to treat the disease. Macrophages are important target cells during natural DENV infection of humans. Here, we unraveled the importance of miRNAs in DENV replication in human primary macrophages. The expression profile of miRNAs was determined in truly DENV-infected cells and cells that were exposed but not productively infected by the virus by RNA sequencing. We revealed that only five miRNAs are regulated in primary macrophages challenged with DENV. These results show that miRNAs do not play a major role in DENV replication. Unexpectedly, we did identify a miRNA with moderate yet significant antiviral properties to DENV. Moreover, miRNA-3614-5p was found to not only decrease DENV but also West Nile virus infectivity. Mass spectrometry and bioinformatics analysis identified adenosine deaminase acting on RNA 1 (ADAR1) as one of the targets. Moreover, ADAR1 was observed to promote the early stages of DENV replication. Collectively, our study broadens the knowledge of the contribution of human miRNAs in shaping the network of interactions between DENV and its human host cells.

## Introduction

Dengue, the arboviral disease with the highest incidence worldwide, is caused by dengue virus (DENV). There are four antigenically distinct serotypes of DENV (DENV-1 to -4) which co-circulate in endemic areas [1]. Annually, an estimated 390 million individuals are infected of which 96 million develop a clinically apparent disease [2]. Disease manifestations range from a mild self-limiting febrile illness to life-threatening severe dengue characterized by plasma leakage, hemorrhages and organ impairment [3]. The majority of individuals with severe disease have a heterologous secondary DENV infection [4]. Although DENV vaccination reduces the incidence of severe disease, there are concerns regarding the overall efficacy and safety of the first licensed DENV vaccine [5]. Furthermore, there are no specific antiviral therapies available to treat the disease.

DENV belongs to the family *Flaviviridae*, which encompasses re-emerging arboviruses such as Zika and West Nile viruses (WNV). In humans, DENV replicates predominantly in dendritic cells, monocytes, macrophages and hepatocytes [6]. Upon infection of cells, the 10.7 Kb positive-sense, single stranded RNA genome is translated into a single polyprotein. Thereafter, the polyprotein is cleaved into three structural proteins (capsid, prM and E) and seven non-structural proteins (NS1, NS2A, NS2B, NS3, NS4A, NS4B and NS5) by viral and cellular proteases [7]. The non-structural viral proteins will, subsequently, in companion with several cellular proteins, start negative strand RNA synthesis. The negative RNA strand serves as a template for the synthesis of new positive RNA strands, which then initiate a new translation cycle or associate with capsid proteins to form a nucleocapsid [8]. Virus assembly starts by budding of a newly formed nucleocapsid into the endoplasmic reticulum lumen, thereby acquiring a lipid membrane with prM and E. Progeny virions mature on the trans-Golgi compartment and exit from the cell by exocytosis [8].

Many cellular proteins have been described to aid in DENV replication [9–12]. For example, adenosine deaminase acting on RNA 1 (ADAR1) has been identified as a proviral factor for DENV replication [13,14]. ADAR1 catalyzes the hydrolytic deamination of adenosine to produce inosine in dsRNA substrates, a process known as A-to-I RNA editing. ADAR enzymes can, however, also influence viral replication in an edit-independent manner [15,16]. The mechanism by which ADAR1 promotes DENV replication remains to be elucidated.

How microRNAs (miRNAs) participate in DENV replication is, however, poorly understood. miRNAs are a class of small non-coding RNAs regulating post-transcriptional expression of genes. miRNAs target mRNAs in a sequence-specific manner leading to mRNA degradation or translational repression [17]. It has been estimated that more than 60% of all mammalian mRNAs are targeted by miRNAs [18] and as a consequence, miRNAs contribute to many cellular processes like cell survival, proliferation and differentiation [19]. In addition, miRNAs are part of the intricate network of interactions between pathogens and host cells [20,21]. Indeed, DENV-challenged cell lines were recently shown to have an altered miRNAome compared to non-infected cells [22–25]. For example, increased expression of miR-30e* was observed in DENV-infected U937 and Hela cells. MiR-30e* was found to act as a restriction factor by promoting interferon (IFN)-β production through the NF-κB pathway [23]. On the other hand, miR-146a is upregulated in DENV-infected human monocytes and miR-146a overexpression resulted in increased DENV replication by damping IFN production [24]. Furthermore, we observed downregulation of miR-133a in DENV-infected Vero cells [25]. This was associated with increased expression of polypirimidine tract binding protein, a protein involved in DENV genome replication [25].

In the present study, we evaluated the miRNAome of DENV-challenged human primary monocyte-derived macrophages (MDMs). Furthermore, we differentiated between DENV-positive and DENV-negative cells in the challenged cell population. MDMs were chosen as macrophages represent important target cells for virus replication during natural infection in humans [26]. Five miRNAs were differentially expressed between DENV-positive and DENV-negative cells. Of these, miR-3614-5p is upregulated in DENV-negative cells and its overexpression reduced DENV infectivity. Subsequent proteomic and biochemical analysis revealed ADAR1 as one of the targets of miR-3614-5p. Taken together, DENV replication is influenced by miR-3614-5p, a miRNA that targets the DENV proviral protein ADAR1.

## Methods

### Cell culture

Baby hamster kidney cells clone 21 (BHK-21; ATCC: CCL-110) were cultured in RPMI-1640 supplemented with 10% fetal bovine serum (FBS) (Lonza, Basel, Switzerland), 100U/ml penicillin and 100mg/ml streptomycin (PAA Laboratories, Pasching, Austria). BHK-21 clone 15 cells are not commercially available and were a kind gift from Richard Kuhn (Purdue University). They were grown in Dulbecco’s minimal essential medium (DMEM) (Gibco, the Netherlands) supplemented with 10% FBS, 100U/mL penicillin and 100mg/mL streptomycin, 100 μM of non-essential aminoacids (Gibco) and 10mM of hepes (Gibco). Huh7 cells (JCRB0403) were a kind gift from Tonya Colpitts (University of South Carolina) and were cultured in DMEM/Glutamax supplemented with 10% FBS, 100U/mL penicillin and 100mg/mL streptomycin. Wild-type (WT) mouse embryonic fibroblasts (MEFs), p53 knockout (KO) MEFs (p53^-/-^) and p53/ADAR1 KO MEFs (p53^-/-^ADAR1^-/-^) were a kind gift from Mary A. O’Connell (CEITEC Masaryk University) and were cultured in DMEM supplemented with 10% FBS, 100U/mL penicillin, 100mg/mL streptomycin, 2mM of L-glutamine, 0.1mM of 2-mercaptoethanol (Gibco) and 20mM hepes. C6/36 *Aedes albopictus* cells (ATCC: CRL-1660) were maintained in minimal essential medium (Invitrogen, Carlsbad, California, USA) supplemented with 10% FBS, 25 mM HEPES, 7.5% sodium bicarbonate, 100U/mL penicillin and 100mg/mL streptomycin, 200 mM glutamine, and 100 μM nonessential amino acids. All mammalian cells were cultured at 37°C and 5% CO_2_ and C6/36 cells were cultured at 28°C and 5% CO_2_.

### Generation and characterization of virus stocks

DENV-2 strain 16681 was propagated on C6/36 cells, as described previously [27]. Recombinant GFP-DENV was generated from the infectious clone pFK-DV-G2A strain 16681 (kind gift from Ralf Bartenschlager University of Heidelberg, [28]). The pFK-DV-G2A clone was propagated in *E*. *coli* strain D5α. Upon plasmid purification, the plasmid was linearized with XbaI (New England Biolabs, Ipswich, Massachusetts, USA) and capped RNA transcripts were synthesized by use of an SP6 polymerase (New England Biolabs). Viruses were harvested at 72 hours post-transfection (hpt) of RNA in BHK-21 cells via electroporation (Biorad Gene Pulser Xcell machine; 850 V, 25 μF, no resistance). Thereafter, GFP-DENV was propagated once by infecting C6/36 cells (MOI 0.1). Progeny virions were harvested, aliquoted and snap-frozen at 120 hours post-infection (hpi). WNV strain NY385-99 was a generous gift from Jaap Goudsmit (Crucell Holland BV) and was propagated on BHK-21 cells, as previously described [29]. All virus preparations were characterized, as described before [27,30] by determination of the number of infectious particles by plaque assay on BHK-15 cells and the number of genome-equivalent particles by Q-RT-PCR. UV-inactivated GFP-DENV was obtained by exposure of the virus to UV-light for 4h. Inactivation was confirmed by plaque assay.

### Human macrophage differentiation

Macrophages were obtained by differentiation of human monocytes isolated from peripheral blood mononuclear cells (PBMCs), as described by us before [31]. Briefly, PBMCs were isolated from buffy coats by Ficoll-Paque (GE Healthcare, Hoevelaken, The Netherlands) and monocytes were obtained by adherence to cell culture plates. Monocytes were differentiated to macrophages in presence of recombinant human M-CSF (ProSpec-Tany TechnoGene Ltd, Rehovot, Israel). Monocyte-derived macrophages (MDMs) were characterized by their morphology and the expression pattern CD14^+^, CD80^low^, CD86^+^, CD206^low^ and MHC-II^+^.

### DENV infections

MDMs were mock-infected, challenged with UV-inactivated GFP-DENV (UVi-DENV) or with GFP-DENV. A viral dose of 92 genome equivalents per cell (MOI 10) was used. Infection was allowed for 2 h, after which cells were washed 3 times and fresh RPMI-1640 medium supplemented with 20% FBS and 10ng/ml of M-CSF was added. Cells and supernatants were harvested at the indicated time points. Huh7 and MEF cells were infected at MOIs of 1, 5 and 10 and cells and supernatants were collected at the specified time points.

### Small RNA sequencing

Total RNA was extracted from cells using the mirVana isolation kit (Ambion, life technologies, USA) following the manufacturer’s instructions. cDNA libraries were prepared from 2 μg RNA using the Illumina TruSeq Small RNA sample preparation kit and small RNA indices (Illumina, San Diego, California, USA). Samples belonging to the same donor were pooled and sequencing was performed on a HiSeq 2000 (Illumina) following manufacturer’s instructions with paired 50 base reads plus a 6-base index read. Raw reads were trimmed and de-barcoded by CLC Genomics Workbench (CLC bio, Cambridge, Massachusetts, USA). The miRanalizer platform [32] was used to map reads to known human miRNAs (based on miRBase V.18). Read counts were standardized to reads per million and imported into GeneSpring GX 12.5 PA (Agilent Technologies, Santa Clara, California, USA). Statistical significance (p<0.05) was examined by an ANOVA test and correction for multiple comparisons was performed according to the Benjamini and Hochberg method. Expression profiles were visualized in heat maps using unsupervised clustering analysis with a Pearson correlation using Genesis.

### Transfection of mimics and siRNAs

Huh7 and MEFs cells were seeded in 24-well plates at a cell density of 7.0x10^4^ and 2.0 x10^4^ cells per well, respectively. At 24 h post-seeding, cells were transfected with 1.5μl of lipofectamine RNAi/Max (Invitrogen) and a final concentration of 10mM of miRNAs mimics (Ambion, cat 4464066; miR-3960 (ID: MC22178), miR-4508 (ID: MC21571), miR-4301 (ID: MC17762), miR-181a (ID: MC10381) and miR-3614-5p (ID: MC20080)). A mimic negative control (Ambion, Cat 4464058) was also used. Cells were infected at 24 hpt. The transfection efficiency was assessed by real time PCR. cDNA synthesis and PCRs were performed with the miRCURY LNA universal RT miRNA PCR system (Exiqon, Vedbaek, Denmark) following the manufacturer’s instructions. Primers targeting the mature sequence of the mentioned miRNAs were obtained from Exiqon (LNA PCR primers set). The comparative Ct method was used taking into account the efficiency of the PCR and with miR-16-5p as a reference miRNA. The PCR efficiency was calculated by LinRegPCR (Academic Medical Center, Amsterdam, the Netherlands).

### Cell viability assays

To determine cell viability of transfected and/or infected cells, cells were trypsinized and stained with the ViViD dye (Life technologies), following manufacturer’s instructions.

### Flow cytometry and sorting

At 24 hpi, MDMs were collected and sorted in a Moflo XDP (Beckman coulter) on the basis of GPF expression. Both fractions, GFP-positive and GFP-negative were collected. Also, mock-infected cells were passed through the cytometer and the whole cell population was recovered. The number of infected cells was determined by flow cytometry on the basis of GFP- or E-protein expression. For E staining, cells were permeabilized with 0.5% saponin and stained using the 4G2 antibody (Merk Millipore, Billerica, Massachusetts, USA) and a rabbit anti-mouse IgG coupled to AF647 (Molecular probes, Eugene, Oregon, USA). Flow cytometry was carried out in a LSRII cytometer (BD Biosciences) and analysis was performed with Kaluza 1.1.

### Liquid chromatography and mass spectrometry (LC/MS)

Huh7 cells were transfected with a mimic negative control or with miR3614-5p mimics. At 24 hpt, proteins were extracted with RIPA lysis buffer system (Santa Cruz, Dallas, TX, USA). Proteins were fractionated by SDS-PAGE at 60V for 6 minutes for a total migration length of ~0.5 cm. Gels were rinsed in deionized water and stained overnight with Coomassie G250 (Biorad). Proteins in the entire 0.5 cm gel section underwent standard in-gel tryptic digestion including reduction and alkylation. Peptides were extracted from each gel section and fractionated by a nanoflow reversed-phase ultra-high pressure liquid chromatography system (nanoLC, Dionex) in-line with a Q-Exactive plus mass spectrometer (Thermo Scientific). Peptides were back-flush eluted onto a 50 cm × 75 μm i.d. nanocolumn (Dionex). Samples were analyzed with a 1.5-hr linear gradient (3–50% acetonitrile with 0.1% formic acid), and data were acquired in a data-dependent manner using a top 12 method with a dynamic exclusion of 20 seconds. For data processing, PEAKS 7.5 software (Bioinformatics Solutions Inc., Waterloo, Ontario, Canada) was applied to the spectra generated by the Q-exactive plus mass spectrometer to search against a Human Protein database (trEmble/SwissProt entries) (Uniprot). The false discovery rate was set at 0.1%. Label free quantitation based on the expectation-maximization algorithm was performed in the Q module of PEAKs 7.5. Peptide features and proteins (detected in all 4 samples) were considered significantly different between groups at a fold change ≥1.5 and *p* value ≤ 0.05.

### Pathway and gene ontology enrichment analysis

To further understand the biological relevance of differentially expressed proteins, we performed functional enrichment analysis in the context of the Gene Ontology (GO), Kyoto Encyclopedia of Genes and Genomes (KEGG) and Reactome databases using the ClueGO Cytoscape plugin. A p-value cut-off of 0.001 was used to identify enriched processes. A kappa score was calculated to reflect the relationships between the terms based on the similarity of their associated genes, with the threshold set at 0.3.

### Western blot

Proteins were extracted from cells using the RIPA Lysis Buffer System (Santa Cruz Biotechnology) and the protein concentration was determined via the Bradford assay (Expedeon, Swavesey, UK). Samples (50–90 μg protein) were mixed with 5x Laemmli buffer and heated at 95°C for 5 min for denaturation. Proteins were fractionated by SDS-PAGE and transferred to Polyvinylidene difluoride membranes (Immobilon-P, Millipore, Darmstad, Germany). Blocking was performed with 5% bovine serum albumin (GE Healthcare) for 10 min. Primary antibodies were added overnight at 4°C. The antibody against ADAR1 (Santa Cruz Biotechnology) was diluted 1:1000 and the GAPDH antibody (Abcam, Cambridge, UK) was diluted 1:10000. After extensive washing, membranes were incubated with secondary HRP-conjugated antibodies, anti-mouse or anti-rabbit (Thermo Fisher Scientific), diluted 1:4000. Membranes were extensively washed before detection. Pierce ECL western blotting substrate (Thermo Fisher Scientific) or Super Signal West FEMTO (Thermo Fisher Scientific) was used for detection by means of chemiluminescence using LAS-4000 mini camera system (Fujifilm Life Science Systems, Japan).

### Statistical analysis

All data was analyzed in GraphPad Prism software and is presented as mean ± SEM. The tests used to evaluate statistical differences between treatments are specified in each figure and a *p* value ≤0.05 was considered significant with *p≤0.05 and **p≤0.01.

### Ethics statement

Buffy coats were obtained from adult healthy volunteers with written informed consent from Sanquin blood bank (Groningen, the Netherlands), in line with the declaration of Helsinki. All samples were analyzed anonymously.

## Results

### DENV alters the expression profile of miRNAs in MDMs

Our initial objective was to provide an insight into the miRNAome of primary human MDMs challenged with DENV. Previously, we and others reported that MDMs are permissive to DENV [31,33,34]. However, even upon DENV challenge at high MOI doses, only a fraction of the cells is productively infected [31,33]. Therefore, we here decided to differentiate between infected cells (DENV-plus) and cells that, despite being in contact with the virus, were not infected (DENV-neg). To facilitate this, GFP-encoding recombinant DENV (GFP-DENV) was used in our experiments [28].

**Fig 1:**
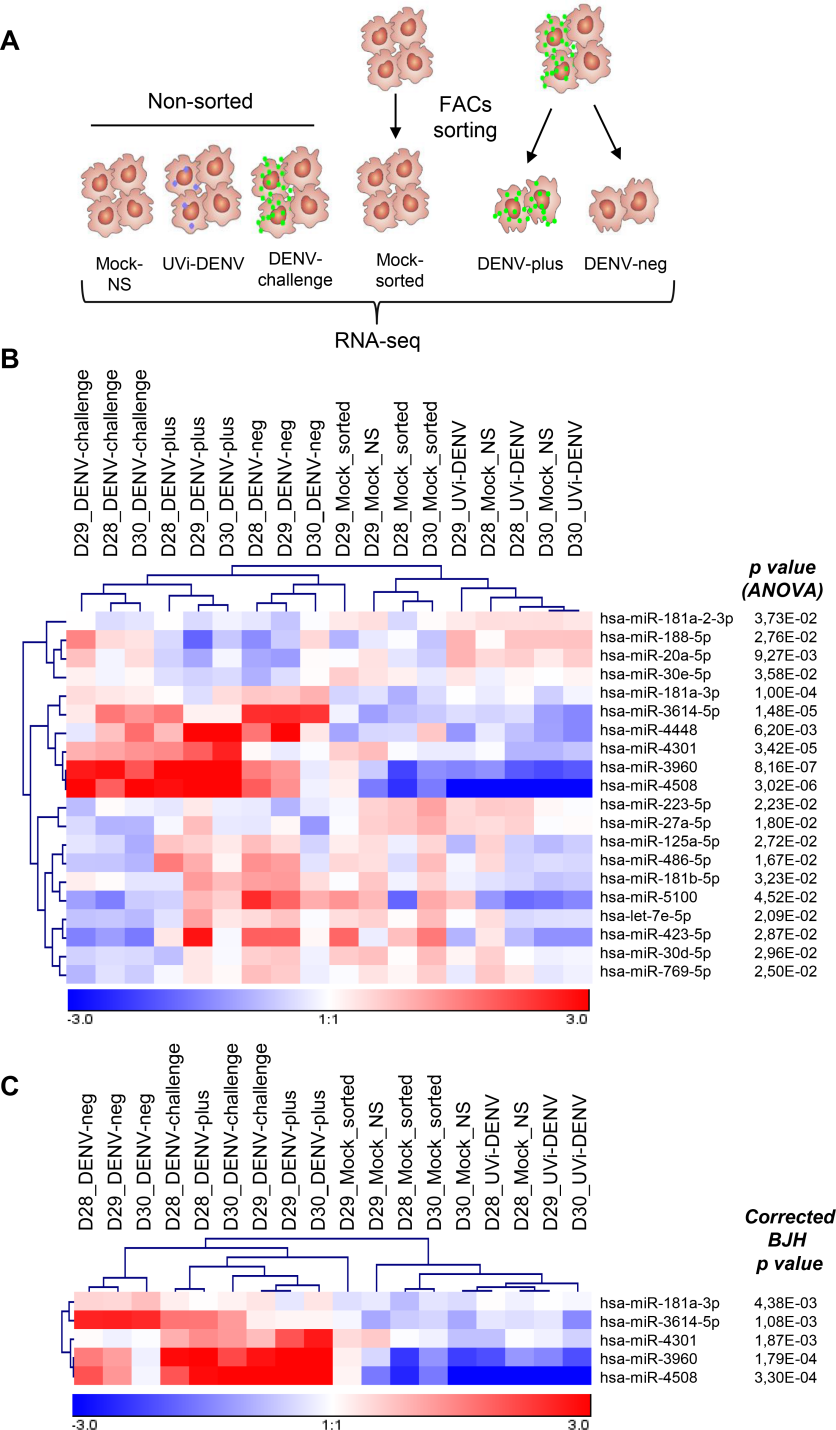
Differentially expressed miRNAs in MDMs challenged with DENV. (A) MDMs obtained from three different blood donors (D28, D29 and D30) were treated as follows: 1) mock-infected and non-sorted, 2) treated with UVi-DENV, 3) challenged with GFP-DENV (DENV-challenge), 4) mock-infected and passed through the FACS sorter, 5) challenged with GFP-DENV and sorted for GFP positive cells (DENV-plus), 6) challenged with GFP-DENV and sorted for GFP negative cells (DENV-neg). (B) Hierarchical unsupervised Pearson correlation of miRNAs showing differentially expressed miRNAs across the samples (ANOVA, p<0.05). (C) Differentially expressed miRNAs across the samples after the Benjamini and Hochberg correction for multiple testing (p<0.05).

Human primary MDMs from three different blood donors, which were cultured independently, were challenged with GFP-DENV at MOI 10 (92 genome equivalents per cell). At these conditions, we anticipate that most of the cells have been in contact with the virus. At 24 hpi, the percentage of infection was determined by means of GFP expression and infectious virus particle production was measured by plaque assay. The percentage of infection was 30.3, 36.6, and 48.4, with corresponding titers of 2.43x10^5^, 1.09x10^5^ and 1.12x10^6^ PFU/ml, respectively (S1 Fig). For small RNA sequencing, DENV-challenged MDMs were sorted in DENV-plus and DENV-neg cell populations and as controls DENV-challenged MDMs without sorting (DENV-challenge), UVi-GFP-DENV (UVi-DENV) and two sets of mock-infected MDMs were added (Fig 1A). One set of mock-infected MDMs were passed through the FACS sorter and compared with non-sorted mock-infected cells to assess the effect of the sorting procedure on miRNA expression. On average, 700 miRNAs were identified per condition, but only those with an average of at least 100 reads per million (in total 219) were taken into account for further analysis. The expression profile of the identified miRNAs is shown in S2 Fig. Notably, most miRNAs cluster on the basis of the donor rather than the condition, highlighting the intrinsic differences that exist among human donors and the limited changes induced by DENV infection.

**Fig 2:**
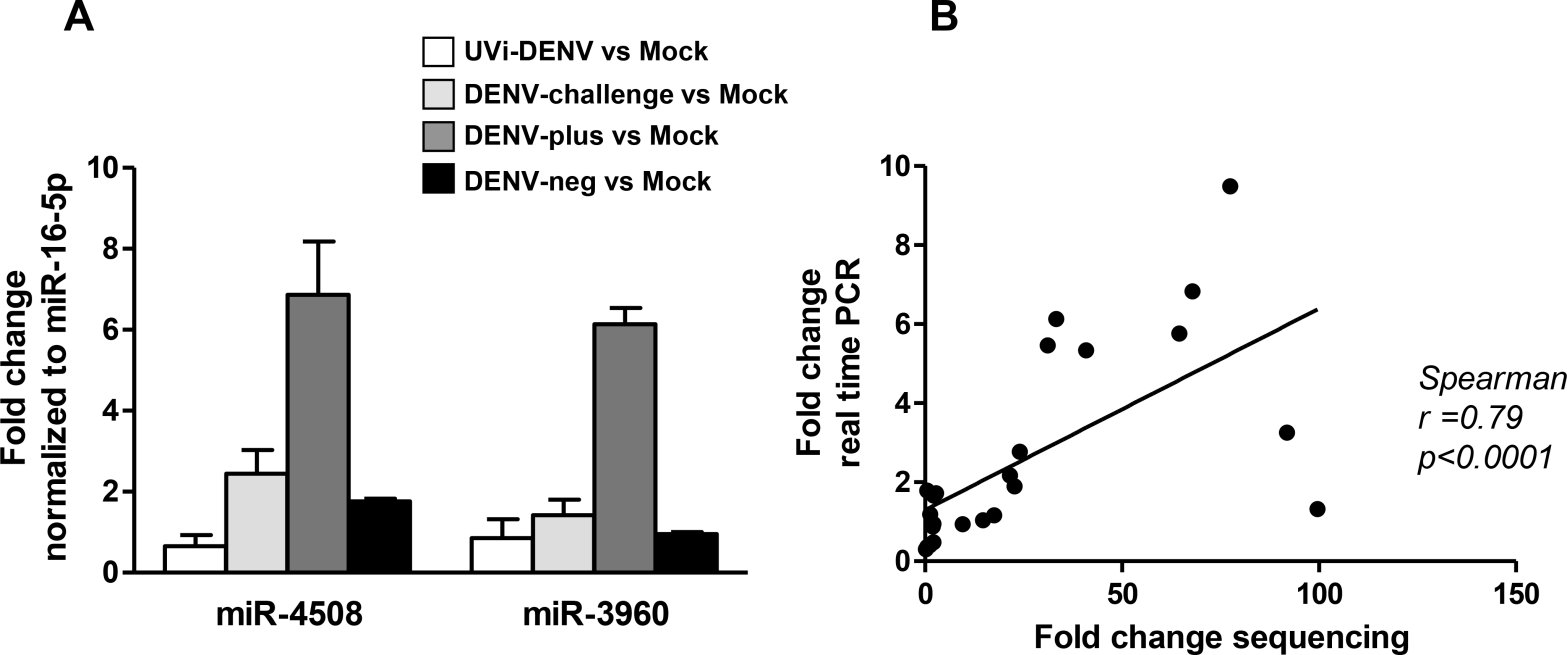
Validation of the miRNA expression profile. (A) miR-3960 and miR-4508 expression was determined by real-time PCR using specific primers for the mature form of the miRNAs. Fold changes of the respective comparisons were determined through the comparative Ct method taking into account the PCR efficiency and miRNA-16-5p as reference miRNA. Data is presented as mean ± SEM from three different blood donors. (B) Spearman correlation between the fold changes obtained by RNA sequencing and real-time PCR.

In order to detect miRNAs differentially expressed, we first compared the two sets of mocks and found no differences between mock-infected cells with and without FACS sorting, demonstrating that the sorting procedure did not affect miRNA expression (S3 Fig). For this reason, we included the two sets of mocks in the following analysis. Only 20 miRNAs were differentially expressed between all treatments (mock non- sorted, UVi-DENV, DENV-challenge, mock sorted, DENV-plus and DENV-neg (Fig 1B, ANOVA, *p*<0.05)). Upon multiple testing correction using the Benjamini and Hochberg method, 5 of the miRNAs remained significant (Fig 1C). The Tukey post hoc test revealed that in comparison to the mock-infected cells, miR-4508, miR-3960, miR-3614-5p, miR-181a-3p are upregulated in DENV-challenged cells; miR-4508, miR-3960, and miR-4301 are upregulated in the sorted DENV-plus cell population; and miR-4508, miR-3960, miR-3614-5p and miR-181a-3p are upregulated in the DENV-neg population. Furthermore, miR-3614-5p and miR-181a-3p were significantly upregulated in the DENV-neg cell population when compared to the DENV-plus population. No differences were found between UVi-DENV cells and mock-infected cells (Table 1), indicating that viral replication is required for changes in the miRNAome. To validate the sequencing results, we next analyzed the expression level of the miRNAs with the highest changes (miR-4508 and miR-3960) by real-time PCR. This was assessed in a new set of three donors following the same infection scheme. The results show a 6.9- and 6.1-fold change in miR-4508 and miR-3960 expression levels in DENV-plus cells over mock-infected cells (Fig 2A). In DENV-neg cells, a 1.8 fold change was seen for miR-4508 and no change in miR-3960 was observed when related to mock-infected cells (Fig 2A). The correlation between the two data sets (sequencing and PCR) is statistically significant (Spearman correlation, p< 0.05, Fig 2B) thereby validating the small-RNA seq-based expression profile.

**Table 1:** Fold changes of each miRNA between the indicated comparisons.

miRNA	UVi-DENV vs Mock	DENV-challenge vs Mock	DENV-plus vs Mock	DENV-neg vs Mock	DENV-plus vs DENV-neg
**hsa-miR-4508**	2.73	28.85	103.43	9.04	11.44
**hsa-miR-3960**	1.55	14.41	44.07	4.2	10.49
**hsa-miR-4301**	1.41	1.95	4.05	-1.05	4.24
**hsa-miR-3614-5p**	1.03	3.07	2.35	9.18	-3.91
**hsa-miR-181a-3p**	-1.29	1.53	1.33	1.96	-1.47

Red and blue colors indicate statistical significant differences in the indicated comparison as determined by the Tukey post hoc test. Red: upregulated miRNAs, blue: downregulated miRNAs.

### miR-3614-5p overexpression reduces DENV replication

**Fig 3:**
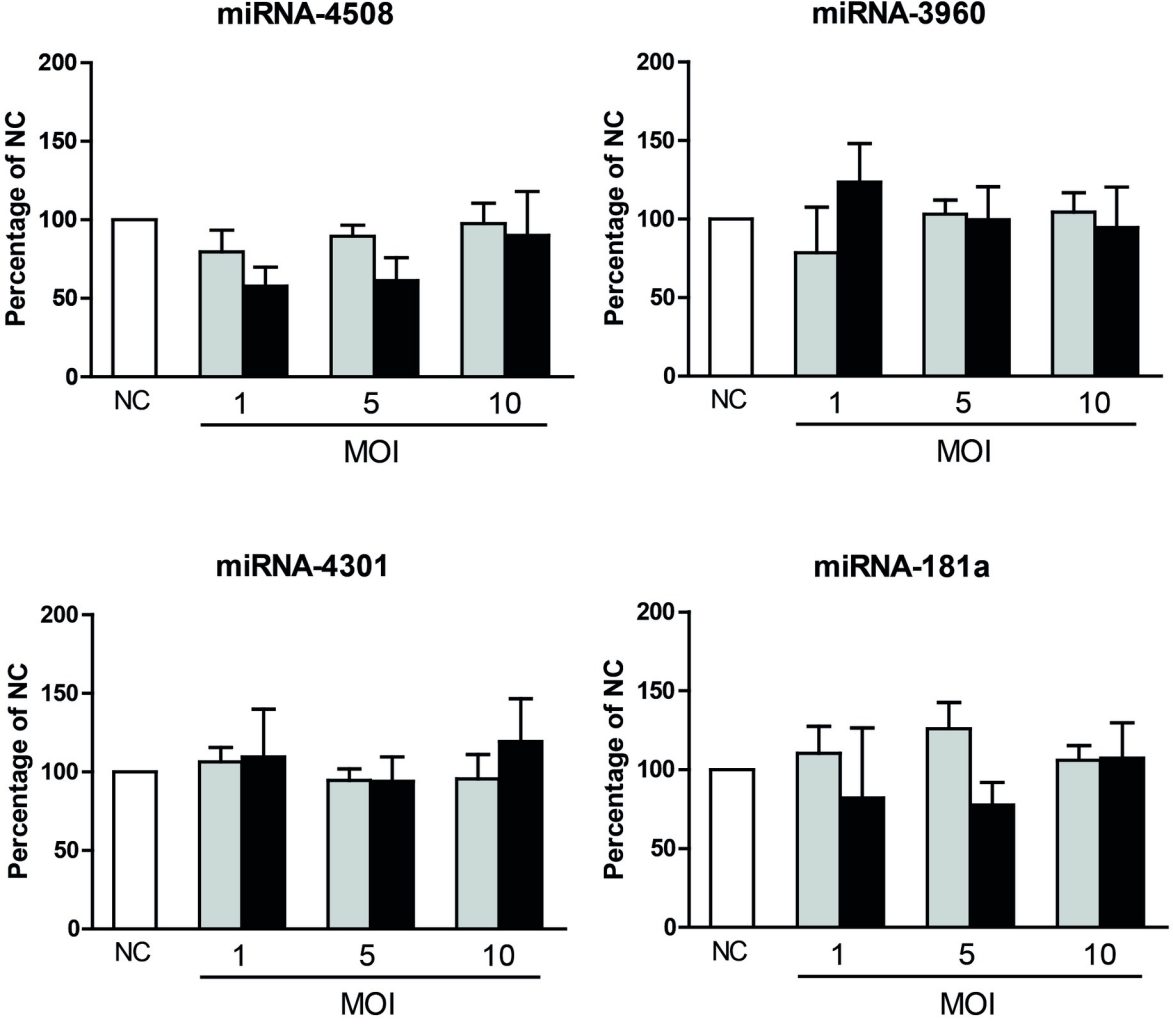
Effect of miR-181a, miR-4301, miR-3960 and miR-4508 on DENV infection of Huh7 cells. Huh7 cells were transfected with a mimic negative control (NC) and with the indicated miRNA mimics. At 24 hpt, cells were infected at MOIs 1, 5 and 10. The percentage of E-positive cells (grey bars) and infectious virus particle production (black bars) were determined at 24 hpi. The percentages of infection of cells transfected with the NC and infected at MOIs 1, 5 and 10 were 6.57±2.07; 13.9±3.48 and 18.53±2.64 respectively; while the viral titers were 1.2x10^5^±5x10^4^; 1.08x10^6^±2x10^5^ and 7.06 x10^6^±6x10^4^ PFU/ml respectively. Data is presented as the percentage relative to the NC and shows mean ± SEM from three independent experiments. Statistical differences were assessed with Student’s t-test.

**Fig 4:**
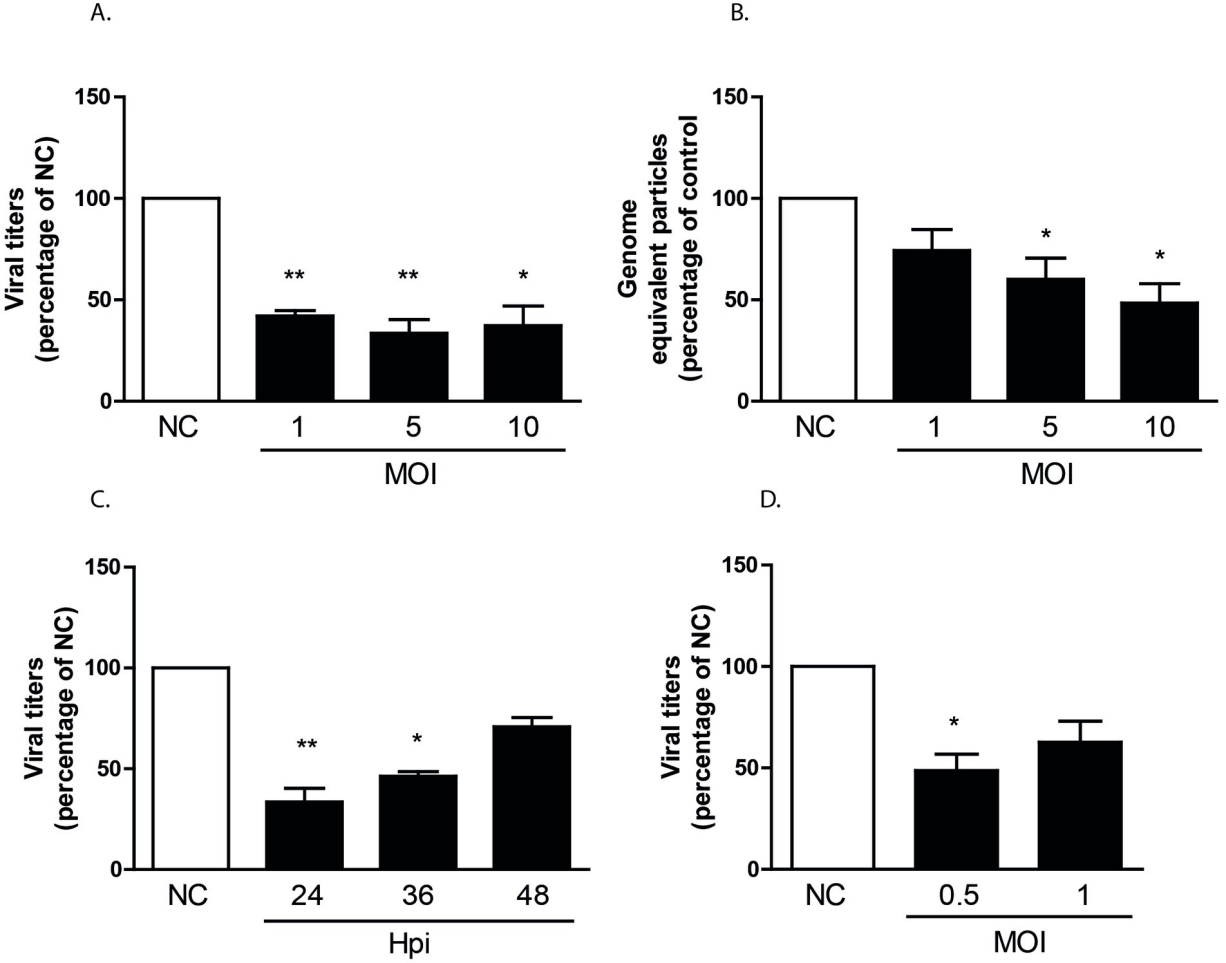
Effect of miR-3614-5p on DENV and WNV infection of Huh7 cells. Huh7 cells were transfected as described in the legend to Fig 3. (A) Infectious virus particle production at 24 hpi. In cells transfected with NC and infected at MOI 1, 5 and 10 the viral titers were 1.2x10^5^±5x10^4^; 1.08x10^6^±2x10^5^ and 7.06x10^6^±6x10^4^ PFU/ml respectively. (B) Number of released DENV genome equivalent particles at 24 hpi. For NC-transfected cells infected at MOIs 1, 5 and 10 the DENV genome equivalent particles per ml were: 2.33x10^7^±1x10^7^; 6.77x10^7^±3x10^7^ and 1.72x10^8^±5x10^7^ respectively. (C) Infectious DENV production following infection at MOI 5 and harvesting times of 24, 36, 48 hpi. The corresponding viral titers of cells transfected with NC were: 1.08x10^6^±2x10^5^; 2.59x10^6^±1x10^6^ and 2.6x10^6^±1x10^6^ PFU/ml. (D) Infectious WNV production at 12 hpi; in cells transfected with the NC the viral titers were 1.82x10^5^±1x10^4^ and 4.14x10^5^±1.32x10^5^ PFU/ml in upon infection at MOIs 0.5 and 1, respectively. Data is presented as the percentage relative to the NC and shows mean ± SEM from at least three independent experiments. Statistical differences were assessed with Student’s t-test.

Small RNA-Seq analysis revealed that only a limited number of miRNAs are regulated in DENV challenged MDMs. To evaluate whether the identified miRNAs influence DENV infectivity, progeny DENV production was determined in MDMs prior transfected with miR-3960, miR-4508, miR-4301, miR-181a, miR-3614-5p, or a negative control (NC) mimic. Although the transfection of the miRNA mimics into MDMs was feasible, the transfection of the NC mimic had a large negative impact on the percentage of infection (S4A Fig). These results forced us to test other cell lines and eventually we found that transfection of Huh7 hepatic human cells with NC mimics had no effect on DENV infectivity and cell viability (S4B and S4C Fig). Furthermore, hepatocytes represent natural target cells for DENV replication [26,35]. Unexpectedly, neither the percentage of infection nor the viral titers were affected by miR-3960, miR-4508, miR-4301 and miR-181a in Huh7 cells following infection at MOIs 1, 5, and 10 (Fig 3). On the contrary, viral titers were significantly reduced (on average 2.8 fold) at all MOIs tested in Huh7 cells overexpressing miR-3614-5p (Fig 4A). In line with these results, a subtle decrease in the number of released genome equivalent viral copies (Fig 4B) was observed. The inhibitory effect of the miRNA on infectious virus particle production is still detectable at 36 h but wanes thereafter (Fig 4C). We next investigated whether miR-3614-5p is expressed in Huh7 cells with and without DENV challenge at MOIs 0.1 and 1 at 12, 24 and 48 hpi. The expression level of miR-3614-5p was found below the detection limit in all experimental conditions tested. The absence or low expression level of miR-3614-5p in Huh7 cells actually make these cells a more astringent model to assess the contribution of miR-3614-5p during DENV infection. To investigate whether the observed effect of miR-3614-5p can be extrapolated to other flaviviruses, we next evaluated the influence of miR-3614-5p on WNV infectivity. WNV infection of miRNA-transfected Huh7 cells was performed at MOIs 0.5 and 1 and viral titers were determined at 12 hpi as WNV is highly infectious in Huh7 cells and replicates faster than DENV. Overexpression of miR-3614-5p in Huh7 cells reduces WNV infectivity to a similar extent as DENV (Fig 4D), indicating that miR-3614-5p has an antiviral effect on both flaviviruses.

### Proteomic changes induced by miR-3614-5p

**Fig 5:**
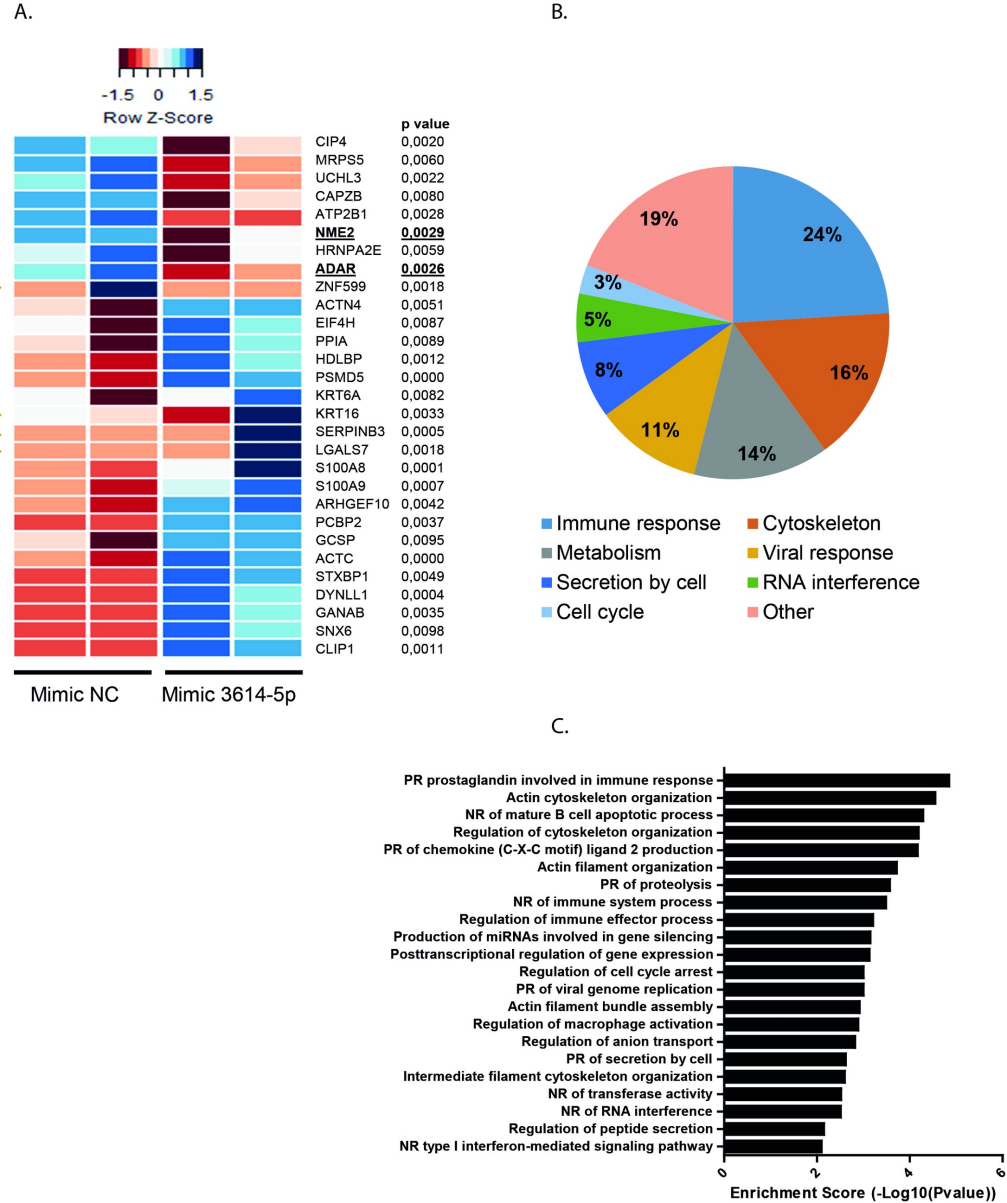
Overexpression of miR-3614-5p regulates protein expression in Huh7 cells. Huh7 cells were transfected with a mimic NC and with the mimic of miR-3614-5p. At 24 hpt changes in protein expression were analyzed by LC/MS. (A) Clustered heat map displaying differentially expressed proteins between the groups (two independent experiments per group). Underlined proteins were also predicted by bioinformatics tools. (B) Gene set enrichment analysis of the proteins regulated by the miR-3614-5p. (C) Enriched KEGG pathways upregulated by the miR-3614-5p (PR: positive regulation, NR: negative regulation).

Based on the above observations, it is likely that miR-3614-5p negatively influences the expression of proteins and/or pathways that promote DENV/WNV replication in Huh7 cells. In order to test this possibility, we first searched for *in silico* predicted targets of miR-3614-5p using the bioinformatics tool miRWalk (which allows a simultaneous search in miRanda, RNA22 and TargetScan) [36]. The identified targets are listed in S1 Table. In total, 694 potential targets were predicted by the four databases. We decided to further identify targets of miR-3614-5p by mass spectrometry, for two reasons; first, computational algorithms have been described to miss real targets and predict large numbers of false positives [37,38] and second, they do not address the context dependency of miRNA/mRNA interactions. In total 2,842 proteins were identified by label free LC/MS (S2 Table). Of these, 29 proteins were significantly differentially expressed with a minimum fold change of 1.5 between NC and miR-3614-5p-overexpressing cells (Fig 5A). Notably, 9 proteins were downregulated and 20 proteins were upregulated in cells overexpressing miR-3614-5p. Furthermore, of these 9 proteins, only ADAR1 and isoform 3 of nucleoside diphosphate B (NME2) were predicted by bioinformatics tools (underlined/bold in Fig 5A), ratifying the aforementioned difficulties of computational methods. ADAR1 was predicted by all four algorithms (miRWalk, miRanda, RNA22 and TargetScan) and NME2 was predicted only by RNA22.

Due to the action of miRNAs, it is likely that the 9 downregulated proteins are direct targets of miRNA-3614-5p. In contrast, the upregulation of the remaining proteins is likely the result of an indirect effect of miR-3614-5p. A gene-set enrichment analysis with particular attention to GO biological processes and KEGG pathways was performed to better understand the involvement of the 29 deregulated proteins in DENV infectivity. As shown in Fig 5B and 5C and S3 Table, the majority of the differentially expressed proteins are associated with the immune response and the organization of the cytoskeleton, two pathways that viruses hijack in order to promote infection. In addition, four proteins were associated with viral response: ADAR1, peptidyl prolyl cis-trans isomerase A (PPIA), serpin B3 (SERPINB3) and isoform 4 of poly(rC)-binding protein 1 (PCBP2).

### miR-3614-5p mediates ADAR1 downregulation

**Fig 6:**
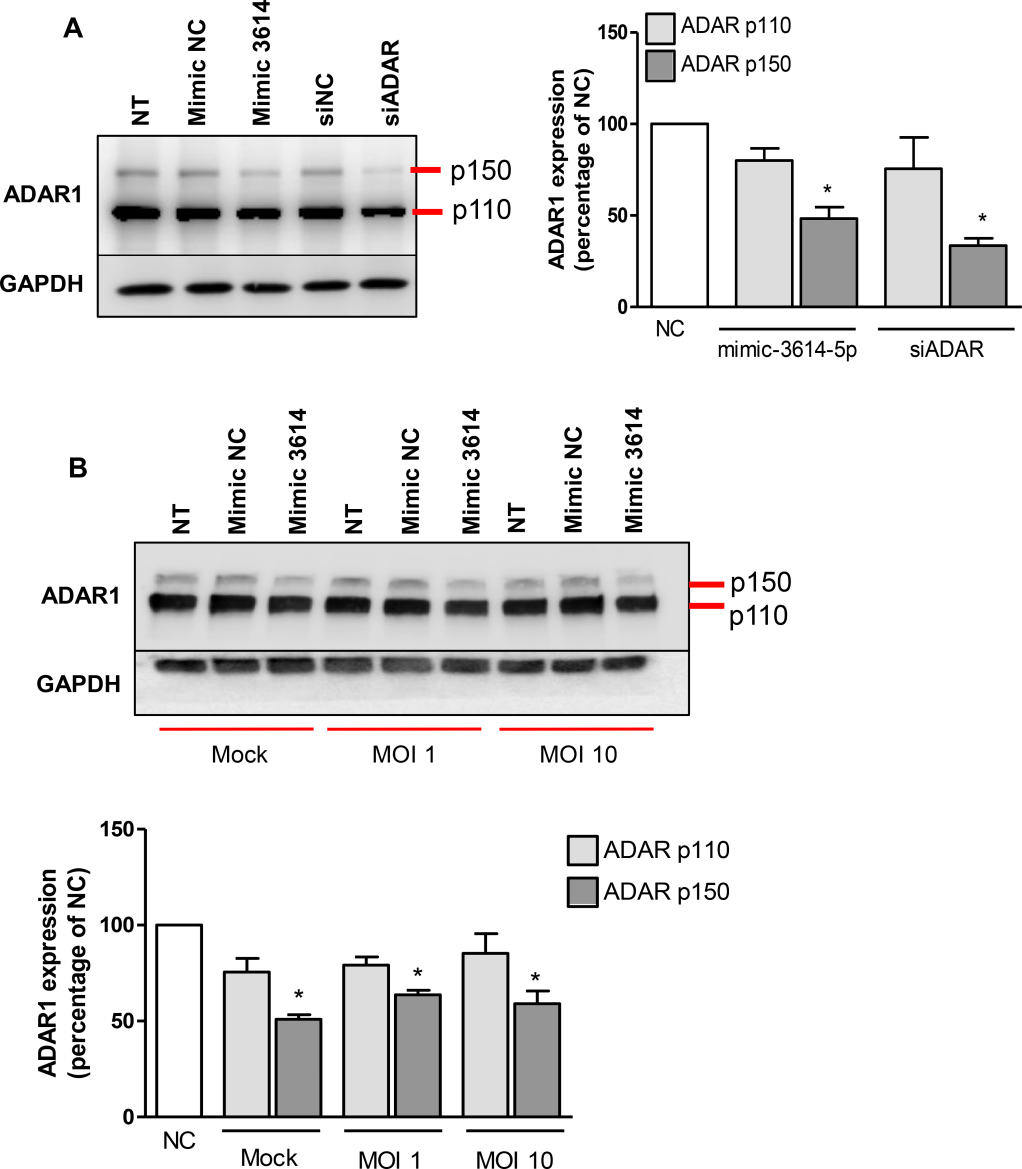
Overexpression of miR-3614-5p downregulates ADAR1 expression in Huh7 cells. (A) Huh7 cells were transfected with the mimic of miR-3614-5p or a siRNA against ADAR1 (siADAR). The correspondent negative control (NC) mimic and NC siRNA were also used. At 24 hpt, total protein was extracted and ADAR1 expression was detected by western blot. (B) Huh7 cells were transfected with the mimic of miR-3614-5p and NC. At 24 hpt, cells were infected with DENV at MOI 1 and 10. At 24 hpi, total protein was extracted and ADAR1 expression was detected by western blot. (A, B) The expression of ADAR1 was normalized to that of GAPDH and it is expressed as the percentage of the cells transfected with the correspondent NC. Data shows mean ± SEM from three independent experiments. Differences were assessed with Student’s t-test.

Our results revealed that multiple proteins are potentially regulated by miR-3614-5p. We considered ADAR1 as an interesting candidate for further validation as ADAR1 is 1) predicted *in silico* as a target by the four programs used, 2) downregulated upon overexpression of miR-3614-5p in our proteomic analysis, and 3) described as a proviral factor for several viruses including DENV [13,14]. There are two isoforms of ADAR1: the constitutively expressed ADAR1 p110 and the interferon (IFN)-induced p150. The p110-isoform accumulates in the nucleus and edits dsRNA before nuclear export. The p150 isoform is active in both the nucleus and the cytoplasm. Furthermore, p150 has been implicated in the editing of viral genomes which impact on the way viruses interact with their hosts, leading to either enhanced or reduced infection [15]. First, we evaluated ADAR1 expression in miR-3614-5p transfected Huh7 cells by western blot analysis. siRNA against ADAR1 was used as a positive control to decrease protein expression. Non-treated Huh7 cells express both, p110 and a basal level of p150 (Fig 6). Interestingly, miR-3614-5p, like siADAR1, reduced the expression of the p150 isoform of ADAR1 (Fig 6A) by 51% and 66%, respectively. No significant differences were found in the protein levels of the p110 isoform at 24 hpt. At 48 hpt, however, siADAR reduced the levels of the p110 isoform by 70% and further decreased the levels of the p150 isoform by 11% (S5 Fig), which is likely due to the higher expression of the p110 isoform. We next determined whether miR-3614-5p also decreases ADAR1 expression in DENV-infected cells. Fig 6B shows that ADAR1 p150 expression is reduced by 36 and 41% in Huh7 cells prior transfected with miR-3614-5p and infected with DENV at MOI 1 and 10, respectively. The moderate but consistent effect of miR-3614-5p on ADAR1 in DENV-infected cells is in line with the subtle effects seen before, thereby strengthening the importance of miR-3614-5p in regulating ADAR1 expression.

### ADAR1 is induced by DENV in MEFs and favors infection

So far our results showed that 1) miR-3614-5p is upregulated in human MDMs that are exposed but not infected with DENV, 2) overexpression of miR-3614-5p reduces DENV infectivity and that 3) ADAR1 p150 expression is repressed by miR-3614-5p in mock-infected and DENV-infected Huh7 cells. In contrast to previously published data [13] we did not observe an increase in ADAR1 p150 in DENV-infected Huh7 cells when compared to mock-infected cells (Fig 6B). On the other hand, we did find an MOI-dependent increase of ADAR1 expression (S6 Fig) in DENV-infected MDMs by microarray analysis [33]. To better understand these results we next decided to use WT MEFs and ADAR KO MEFs as an alternative, more stringent, approach to confirm the role of ADAR in DENV infectivity.

**Fig 7:**
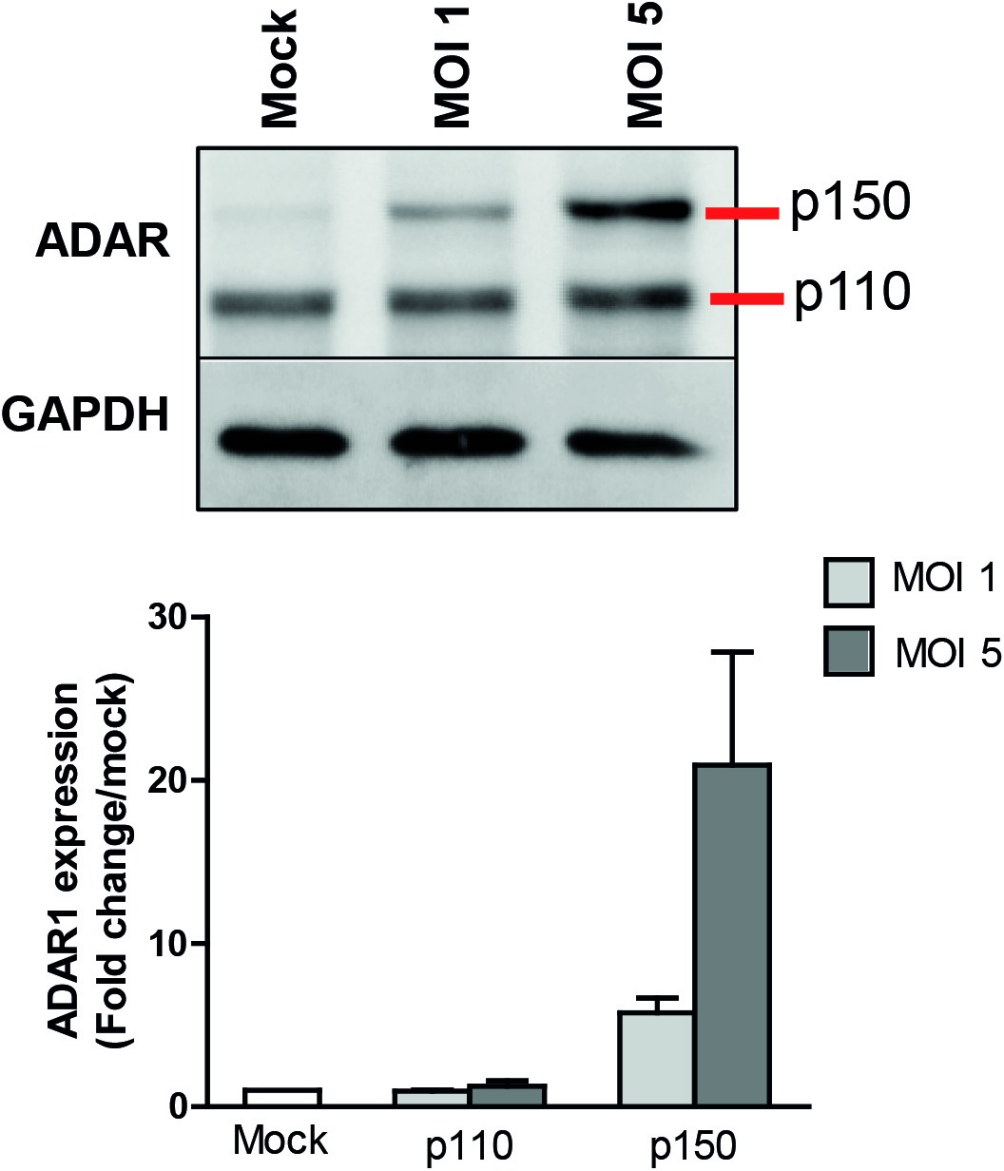
DENV infection induces ADAR1 expression in MEFs. MEFs were infected with DENV at the indicated MOIs. The methodology is as described in the legend to Fig 6. The expression of ADAR1 was normalized to that of GAPDH and it is expressed as the percentage of the mock-infected cells. Data shows mean ± SEM from three independent experiments.

**Fig 8:**
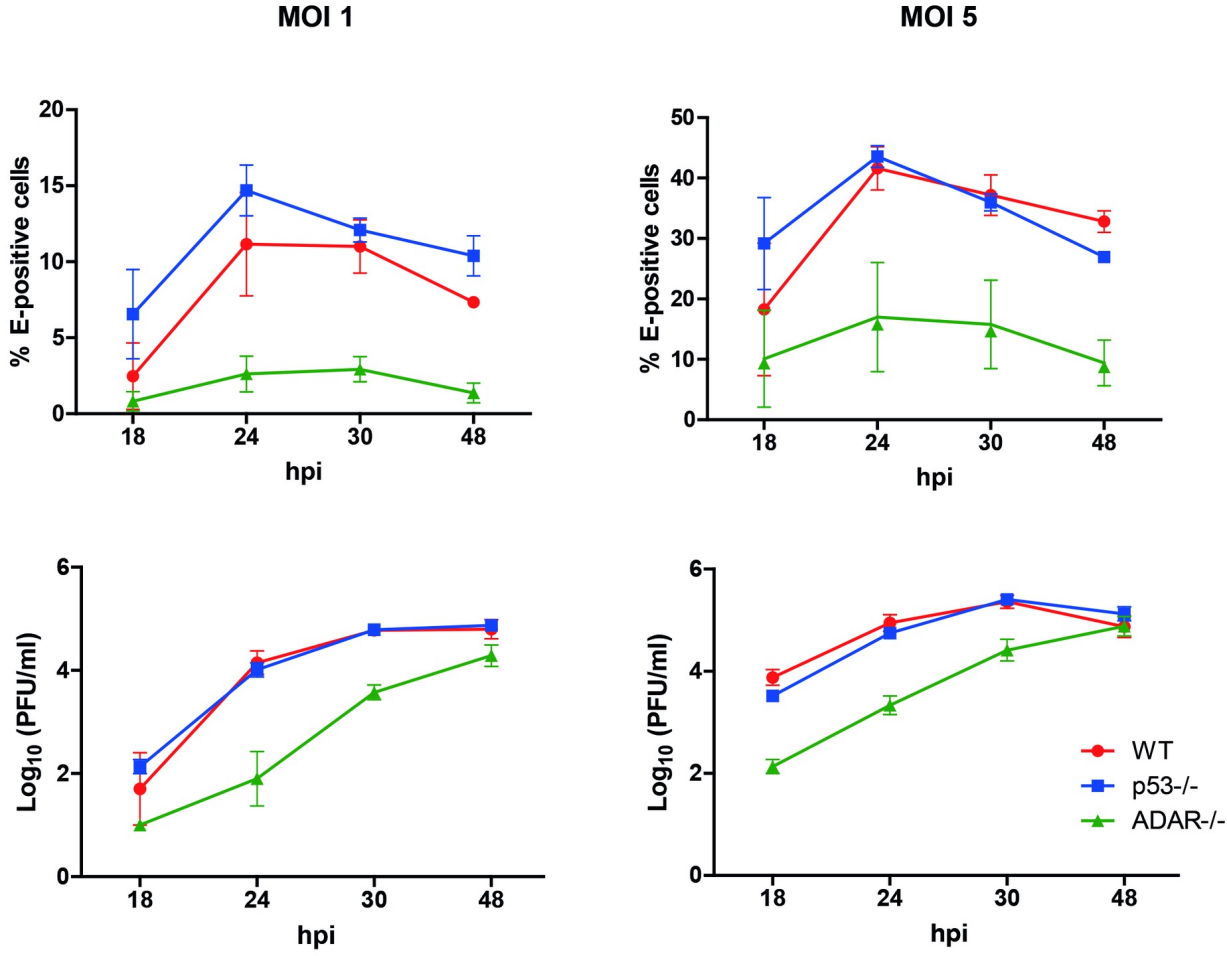
ADAR KO MEFs are less permissive to DENV than wild-type MEFs. Wild-type (WT) MEFs, p53 KO MEFs (p53^-/-^) and p53/ADAR double KO MEFs (ADAR^-/-^) were infected with DENV at MOIs 1 and 5. At the indicated time points, the percentage of infection (upper panels) was determined by flow cytometry and the infectious virus particle production (lower panels) by plaque assay. Data shows mean ± SEM from three independent experiments.

In DENV-infected WT MEFs, an MOI-dependent increase in ADAR1 p150 expression was found. At MOI 1, ADAR p150 increased 5.7-fold and at MOI 5 a 20.9-fold increase was detected (Fig 7). This suggests that ADAR1 expression is indeed enhanced upon DENV infection. We do not have an explanation for our results in Huh7 cells yet it might be related to the high basal levels of ADAR1 in our Huh7 cells as the protein bands are much more intense when compared to WT MEFs under otherwise similar experimental conditions. Next, we investigated whether ADAR1 controls DENV replication. To this end, we used ADAR1 and p53 double KO MEFs as single ADAR1^-/-^ MEFs cannot be cultured long term because of cell death [39]. S7 Fig confirms that p53^-/-^ADAR1^-/-^ cells do not express ADAR1. Furthermore, the expression level of ADAR1 was comparable (not statistically different) in WT and p53^-/-^ MEFs. DENV infectivity was compared between WT, p53^-/-^ and p53^-/-^ADAR1^-/-^ MEFs (Fig 8). The results indicate that both the percentage of infection and the viral titers are significantly lower in the p53^-/-^ADAR1^-/-^ MEFs compared to WT and p53^-/-^ MEFs. The percentage of infected MEFs did not increase beyond 24 hpi, which suggests that MEFs only support one round of replication (Fig 8, upper panels). Infectious virus particle production plateaus at 30 hpi for control MEFs whereas in the p53^-/-^ADAR1^-/-^ cells virus particle production continues till at least 48 hpi (Fig 8, lower panels). At 48 hpi, the number of produced infectious virus particles by the p53^-/-^ADAR1^-/-^ MEFs nearly reaches the levels of control cells despite the fact that the percentage of infected cells at this time point is much lower. Collectively, the data suggests a dual role for ADAR1. ADAR1 appears to contribute to DENV infectivity at early stages of infection, whereas it suppresses long-term virus production.

### miR-3614-5p overexpression reduces DENV replication in WT MEFs but not in ADAR KO cells

**Fig 9:**
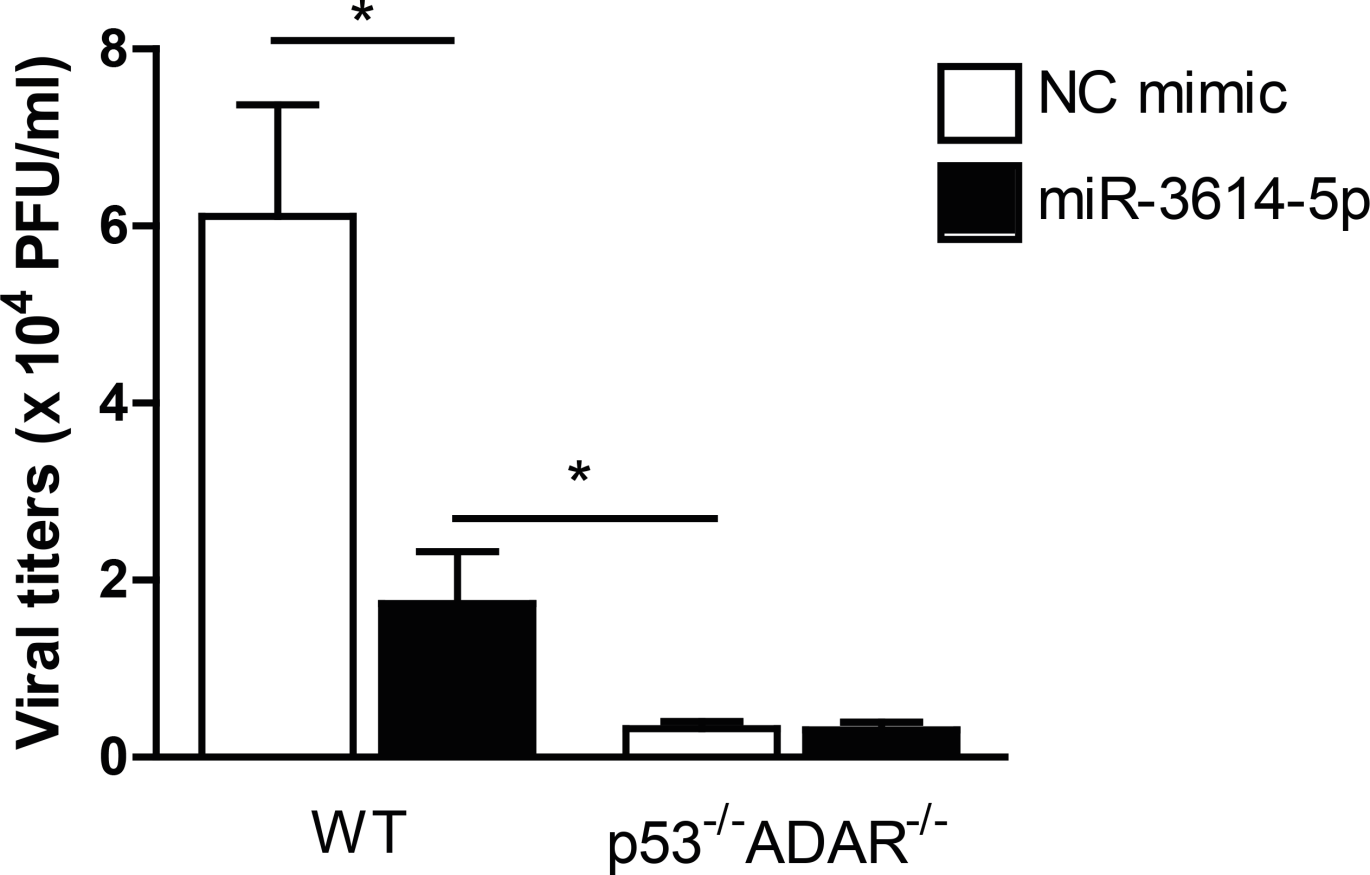
miR-3614-5p impairs DENV production in WT MEFs but not in ADAR KO cells. WT and p53^-/-^ADAR1^-/-^ cells were transfected with the mimic of miR-3614-5p or the correspondent negative control (NC). At 24 hpt, cells were infected with DENV at MOI 5. At 24 hpi, cell supernatants were titrated by plaque assay. Data shows mean ± SEM from three independent experiments. Differences were assessed with Student’s t-test.

We next determined whether overexpression of miR-3614-5p impairs DENV infectivity in WT MEFs and p53^-/-^ADAR^-/-^ MEFs. The results show that transfection of miR-3614-5p in WT MEFs reduced viral titers by 3.7-fold when compared to cells transfected with the mimic negative control (Fig 9). On the other hand, transfection of miR-3614-5p into p53^-/-^ADAR^-/-^ cells did not have an effect on infectious DENV production. This data confirms that miR-3614-5 reduces DENV infection through regulation of ADAR1.

## Discussion

We anticipated that DENV would induce significant changes in the miRNAome of infected cells. However, our results show that only very few miRNAs are regulated in DENV-challenged MDMs. Furthermore, the miRNAs that are specifically upregulated in DENV-infected cells do not impact infectious virus particle production. On the contrary, miR-3614-5p, which was upregulated in DENV-negative MDMs, had a moderate but significant antiviral effect. MiR-3614-5p was observed to downregulate the expression of the IFN-induced p150 isoform of ADAR1. Furthermore, cells that do not express ADAR1 are less permissive to DENV infection. Taken together, through this screen, we identified a novel miRNA with antiviral properties towards two clinically relevant flaviviruses.

Unique to our approach is that we used human primary macrophages and differentiated between truly DENV-infected cells and cells that despite being exposed to virus were not infected. This approach revealed that only 5 miRNAs are differentially expressed. The overall low number of miRNAs that are regulated upon DENV infection is in line with other studies in cell lines. For example, Wu *et*. *al* described that infection of human endothelial cells (EA.hy926) with DENV induced upregulation of 8 miRNAs and downregulation of 4 miRNAs [40]. Similarly, Escalera-Cueto and colleagues reported that 9 miRNAs are regulated upon DENV infection of Huh7 cells [41]. None of the previously reported miRNAs were found in our study, revealing the strong cellular-context dependency of miRNA regulation in response to DENV infection. The low number of miRNAs regulated in DENV-infected cells and the lack of overlap among different cell types strongly suggest that cellular miRNAs do not play a key role during DENV replication.

None of the miRNAs upregulated in the DENV-plus population of MDMs had an impact on DENV infectivity in Huh7 cells. It therefore appears that none of these miRNAs have a universally beneficial effect on DENV replication. We, however, cannot rule out potential proviral functions of these miRNAs in MDMs. Alternatively, their increased expression might be a response to either cellular stress or immune activation induced by the infection [42,43]. For example, an increased expression of miR-4508 and miR-4301 has been observed in several cancer types [44–47] and it was previously reported that miR-3960 can be used as a marker for type 2 diabetes [48]. The observation that these miRNAs can be induced by diverse stimuli, points towards a general mechanism of activation, likely mediated by cellular stress and/or immune responses.

MiR-3614-5p, on the other hand, was found upregulated in DENV-neg MDMs and its overexpression had a moderate but significant negative effect on DENV and WNV infectivity in Huh7 cells. MiR-3614 is not regulated in cells treated with UV-iDENV, suggesting that the expression level of miR-3614 is increased as a consequence of abortive infection or is triggered by soluble factors secreted by infected cells. Although the contribution of miRNAs in regulating replication of mammalian-infecting viruses is highly debated [49,50], we found a consistent reduction of DENV and WNV infectivity. Furthermore, antiviral activity of many other miRNAs in other viral systems have been reported [21,22]. The observed effect of miRNAs on virus replication is generally subtle, though this is expected as miRNAs are fine-tuners of gene expression, reducing protein levels of their targets by on average 2-fold [24,41,51–55]. In addition, the use of redundant host factors by mammalian-infecting viruses could add to the limited effect of individual miRNAs on virus production.

To elucidate the mode of action for miR-3614-5p, we performed proteomic analysis of miR-3614-5p-overexpressing cells. The results revealed that 9 proteins are downregulated and 20 proteins are upregulated in response to miR-3614-5p. Some of the identified proteins have been implicated in the replication cycle of mammalian viruses. For example, protein S100-A9 (S100A9) and α-actinin-4 (ACTN4) were described to enhance influenza A virus infection, and dynein light chain 1 (DYNLL1) has been implicated in the transcription of human immunodeficiency virus [56–58]. Heterogeneous nuclear ribonucleoproteins A2/B1 (HNRNPA2B1) was found to participate in Japanese encephalitis virus replication [59] and was described to bind to the 3’UTR of DENV RNA [60]. A clear role for HNRNPA2B1 during DENV infection was, however, not determined [60,61]. Additionally, ADAR1 has been reported as a proviral factor for multiple viruses [14]. We examined the role of miR3614-5p in regulating ADAR1 expression and showed that miR-3614-5p reduces ADAR1 p150 protein levels in mock-infected and DENV-infected cells. Two distinct methodologies were used in this analysis and therefore it is likely that miR-3614-5p regulates ADAR1 expression. To confirm ADAR1 as a direct target of miR-3614-5p other experiments such as the use of reporter assays might be required. In addition, future research should dissect whether miR-3614-5p- also regulates the expression of the other proteins identified by LC/MS.

De Chassey and colleagues reported that DENV infection of Huh7 cells increases ADAR1 expression. Furthermore, silencing of ADAR1 through siRNAs was found to decrease DENV replication [13]. Although in our system, DENV did not alter the expression of ADAR1 in Huh7 cells, we did observe an increase in ADAR1 levels in MDMs and WT MEFs. We speculate that the discrepancies between previously published data [13] and our results in Huh7 cells, might relate to the basal levels of ADAR1. The higher basal levels of ADAR1 in our Huh7 cells when compared to MEFs is in line with the stronger effect of miR-3614-5p on DENV infectivity observed in MEFs (3.7-fold decrease) when compared to Huh7 cells (2.8-fold decrease). In WT MEFs, DENV infection specifically upregulated the IFN-inducible p150 isoform, suggesting that virus-induced IFN might play an active role in this phenomenon. Furthermore, ADAR1 KO MEFs were less susceptible to DENV infection and initially produced lower numbers of progeny infectious virus particles. At late time points, however, virus particle production per infected cell is much higher in the ADAR1 KO MEFs. Collectively, this strongly suggests that ADAR1 acts as a proviral factor early in replication whereas at late time points it represses DENV replication. DENV infection was shown to increase the overall ADAR1 editing activity [13]. Thus, it is likely that viral RNA is subjected to A-to-I modifications which have been associated with the suppression of innate immune responses, thereby allowing more efficient replication early in infection [39,62,63]. However, the exact mechanism by which ADAR1 promotes and later limits DENV infectivity remains to be elucidated.

We show that miR-3614-5p has antiviral activity towards flaviviruses and regulates ADAR1 expression, yet future studies should unravel whether there is a direct causal link between these findings. Furthermore, it remains to be elucidated whether MDMs are more refractory to infection due to upregulation of miR-3614-5p. Dissecting the molecular actions of miR-3614-5p will deepen our understanding of the replication cycle of flaviviruses and how the expression of miRNAs is regulated in primary human/relevant cell types.

## Supporting information

S1 Fig MDMs are susceptible to the infection by a recombinant GFP-DENV. MDMs from three different donors (D29, D30, D28) were infected at MOI 10 with a recombinant GFP-DENV. At 24 hours post-infection, the percentage of GFP-positive cells was determined by flow cytometry.
(TIF)

S2 Fig Hierarchical unsupervised Pearson correlation of miRNAs detected in MDMs challenged with DENV. MDMs from three different blood donors (D28, D29 and D23) treated as follow: 1) mock-infected, 2) treated with UVi-DENV, 3) challenged with GFP-DENV (DENV-challenge), 4) challenged with GFP-DENV and sorted for GFP positive cells (DENV-plus), 5) challenged with GFP-DENV and sorted for GFP negative cells (DENV-neg).
(TIF)

S3 Fig FACs sorting does not influence the expression of miRNAs in MDMs. (A) Hierarchical unsupervised Pearson correlation of miRNAs detected in MDMs mock-infected non-sorted (NS) and passed through the FACs sorting. (B) Comparison average number of reads per million (RPM) from mock-infected NS and mock-infected sorted cells. No differences were found between the groups when a moderated T test was applied.
(TIF)

S4 Fig Effect of mimics transfection on DENV infectivity and cell viability. Cells were transfected at a final concentration of 10nM of the indicated miRNA mimic or not transfected (NT). At 24 hpt, MDMs (A) and Huh7 (B) were infected at MOIs 1, 5 and 10. At 24 hpi, the percentage of E-positive cells was determined by flow cytometry. Data is presented as the percentage relative to the NT cells and shows mean ± SEM from three different blood donors (A) and at least three independent experiments (B). Differences were assessed with Student’s t-test. (C) At 24 hpt, viability of Huh7 cells was determined. Data shows mean ± SEM from three independent experiments.
(TIF)

S5 Fig Overexpression of miRNA-3614-5p downregulates ADAR1 expression in Huh7 cells. Huh7 cells were transfected with the mimic of miRNA-3614-5p or a siRNA against ADAR1 (siADAR). The correspondent negative control (NC) mimic and NC siRNA were also used. At 48 hpt, total protein was extracted and ADAR1 expression was detected by western blot. The expression of ADAR1 was normalized to that of GAPDH and it is expressed as the percentage of the cells transfected with the correspondent NC. Data shows mean ± SEM from three independent experiments. Differences were assessed with Student’s t-test.
(TIF)

S6 Fig DENV infection induces ADAR1 expression in human MDMs. MDMs were infected with DENV at the indicated MOIs and at 24 hpi total RNA was extracted. Gene expression was investigated by microarray [33]. Probe values were normalized against the total signal intensity of the sample and subsequently, the fold change of the probes were expressed relative to the mock condition of the same donor taking into account the house keeping genes (HKGs) GAPDH, β-actin, β-glucuronidase, Hypoxanthine-guanine phosphoribosyltransferase and heat shock protein 90β1. Data shows mean ± SD from four different blood donors.
(TIF)

S7 Fig ADAR1 expression in wild-type and KO MEFs. Representative blot of ADAR1 expression in cultured wild-type MEFs (WT), p53 KO MEFs (p53^-/-^) and p53/ADAR double KO MEFs (p53^-/-^ADAR^-/-^). The expression of ADAR1 was normalized to that of GAPDH and it is expressed as the percentage of the WT cells. Data shows mean ± SEM from three independent experiments. ND, no determined.
(TIF)

S1 Table List of miR-3614-5p targets predicted by miRWalk. (XLSX)

S2 Table List of proteins identified by mass spectrometry. (XLSX)

S3 Table KEEGG pathways altered by the overexpression of miR-3614-5p. (XLSX)
