# Supplementary figures and images for: MicroRNA profiling of human primary macrophages exposed to dengue virus identifies miRNA-3614-5p as antiviral and regulator of ADAR1 expression
Source: PLoS Negl Trop Dis. 2017 Oct 18;11(10):e0005981. doi: 10.1371/journal.pntd.0005981 (PMC5662241; doi:10.1371/journal.pntd.0005981)

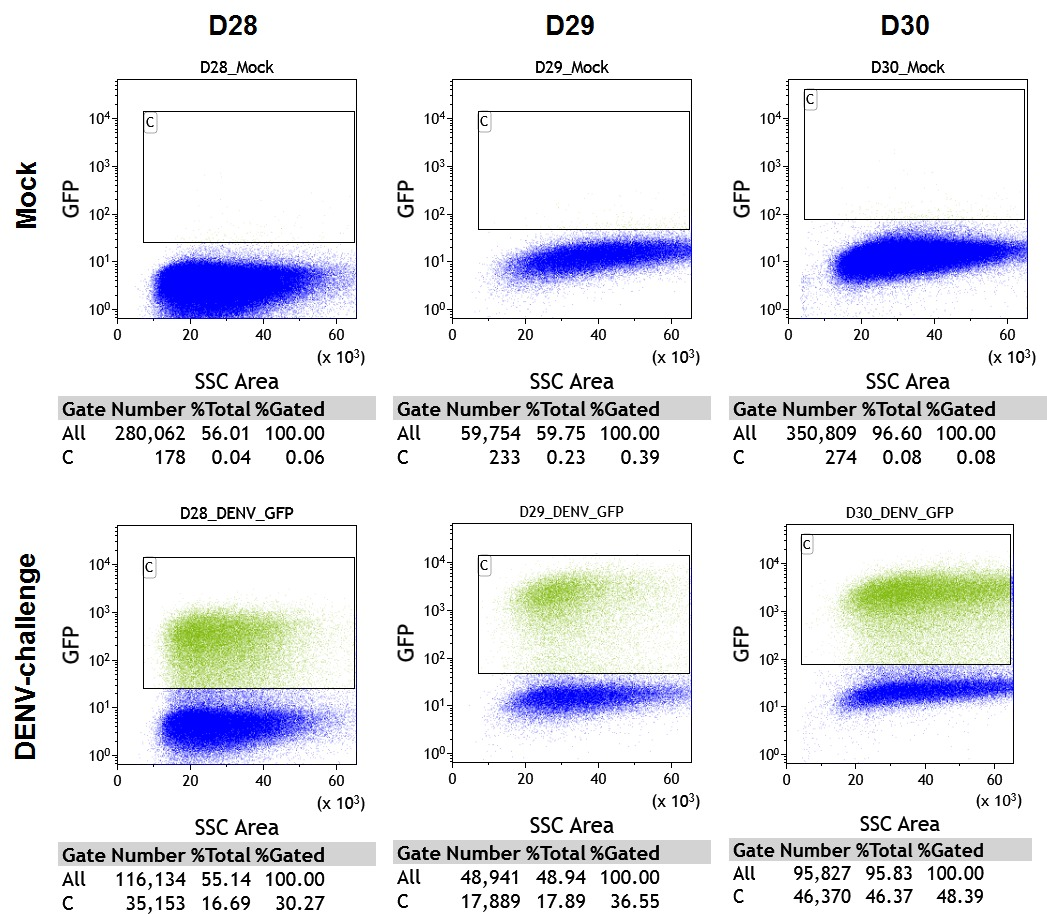

Supplement: S1 Fig — MDMs from three different donors (D29, D30, D28) were infected at MOI 10 with a recombinant GFP-DENV. At 24 hours post-infection, the percentage of GFP-positive cells was determined by flow cytometry. (TIF) [file pntd.0005981.s001.tif]

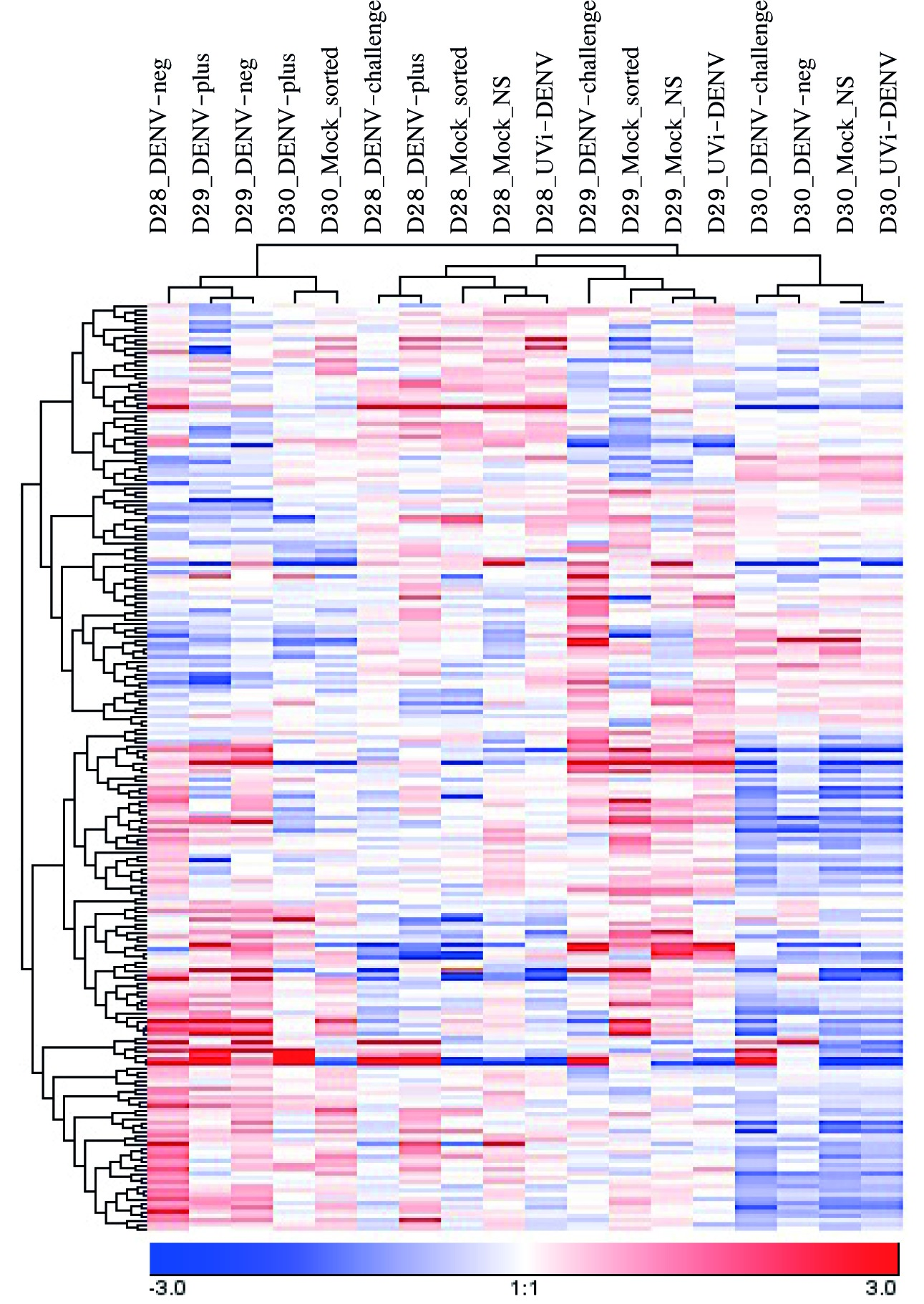

Supplement: S2 Fig — MDMs from three different blood donors (D28, D29 and D23) treated as follow: 1) mock-infected, 2) treated with UVi-DENV, 3) challenged with GFP-DENV (DENV-challenge), 4) challenged with GFP-DENV and sorted for GFP positive cells (DENV-plus), 5) challenged with GFP-DENV and sorted for GFP negative cells (DENV-neg). (TIF) [file pntd.0005981.s002.tif]

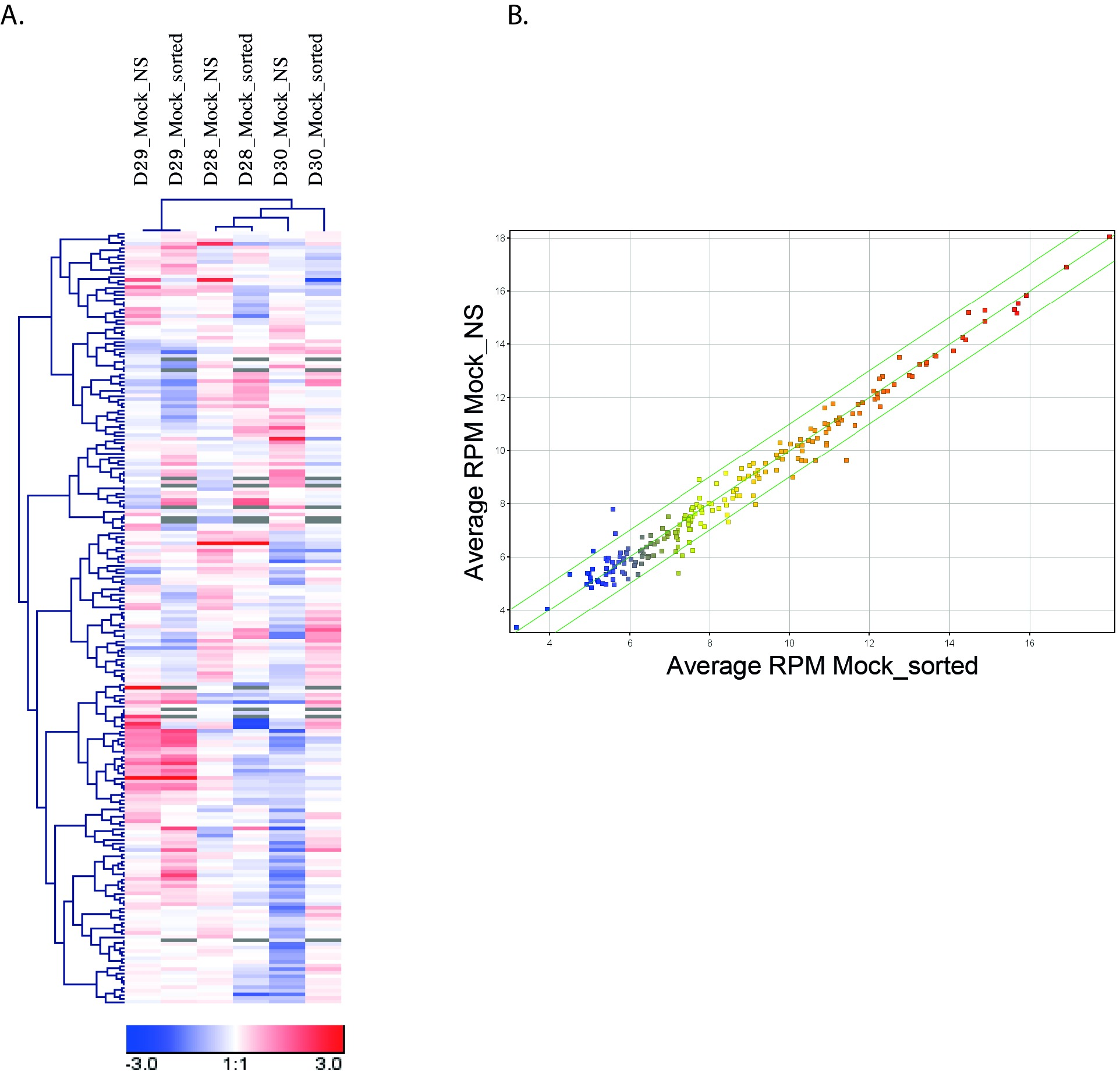

Supplement: S3 Fig — (A) Hierarchical unsupervised Pearson correlation of miRNAs detected in MDMs mock-infected non-sorted (NS) and passed through the FACs sorting. (B) Comparison average number of reads per million (RPM) from mock-infected NS and mock-infected sorted cells. No differences were found between the groups when a moderated T test was applied. (TIF) [file pntd.0005981.s003.tif]

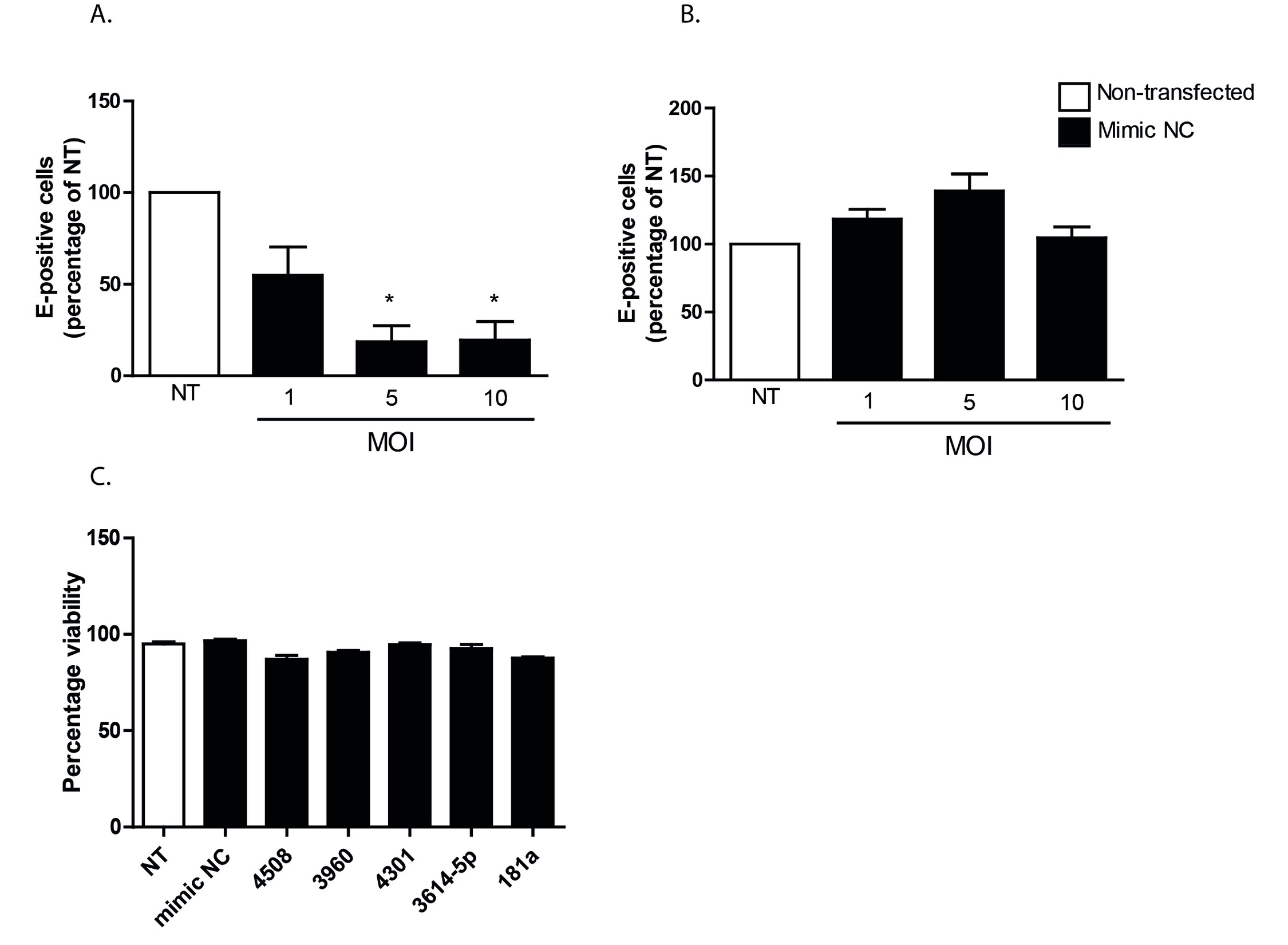

Supplement: S4 Fig — Cells were transfected at a final concentration of 10nM of the indicated miRNA mimic or not transfected (NT). At 24 hpt, MDMs (A) and Huh7 (B) were infected at MOIs 1, 5 and 10. At 24 hpi, the percentage of E-positive cells was determined by flow cytometry. Data is presented as the percentage relative to the NT cells and shows mean ± SEM from three different blood donors (A) and at least three independent experiments (B). Differences were assessed with Student’s t-test. (C) At 24 hpt, viability of Huh7 cells was determined. Data shows mean ± SEM from three independent experiments. (TIF) [file pntd.0005981.s004.tif]

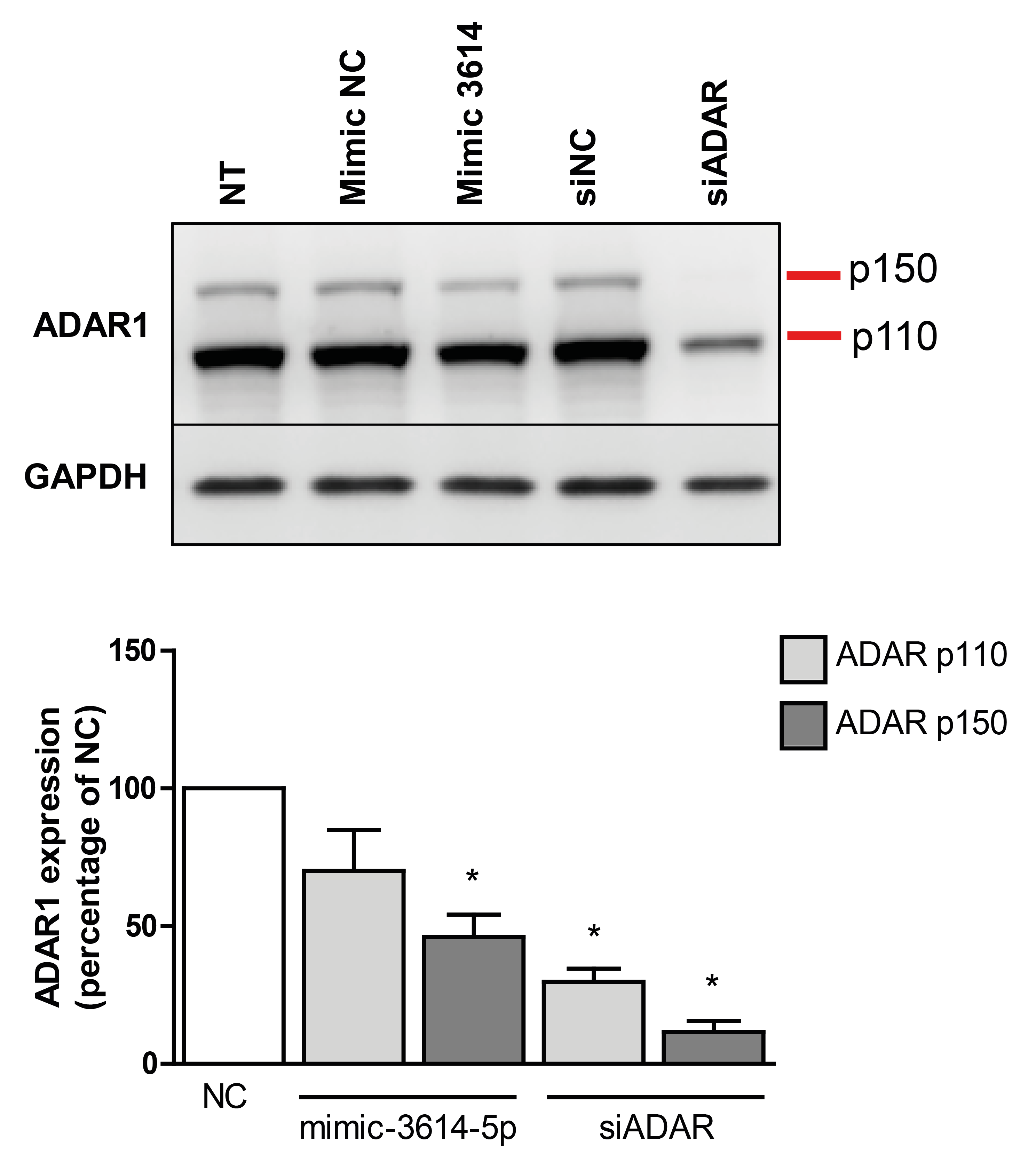

Supplement: S5 Fig — Huh7 cells were transfected with the mimic of miRNA-3614-5p or a siRNA against ADAR1 (siADAR). The correspondent negative control (NC) mimic and NC siRNA were also used. At 48 hpt, total protein was extracted and ADAR1 expression was detected by western blot. The expression of ADAR1 was normalized to that of GAPDH and it is expressed as the percentage of the cells transfected with the correspondent NC. Data shows mean ± SEM from three independent experiments. Differences were assessed with Student’s t-test. (TIF) [file pntd.0005981.s005.tif]

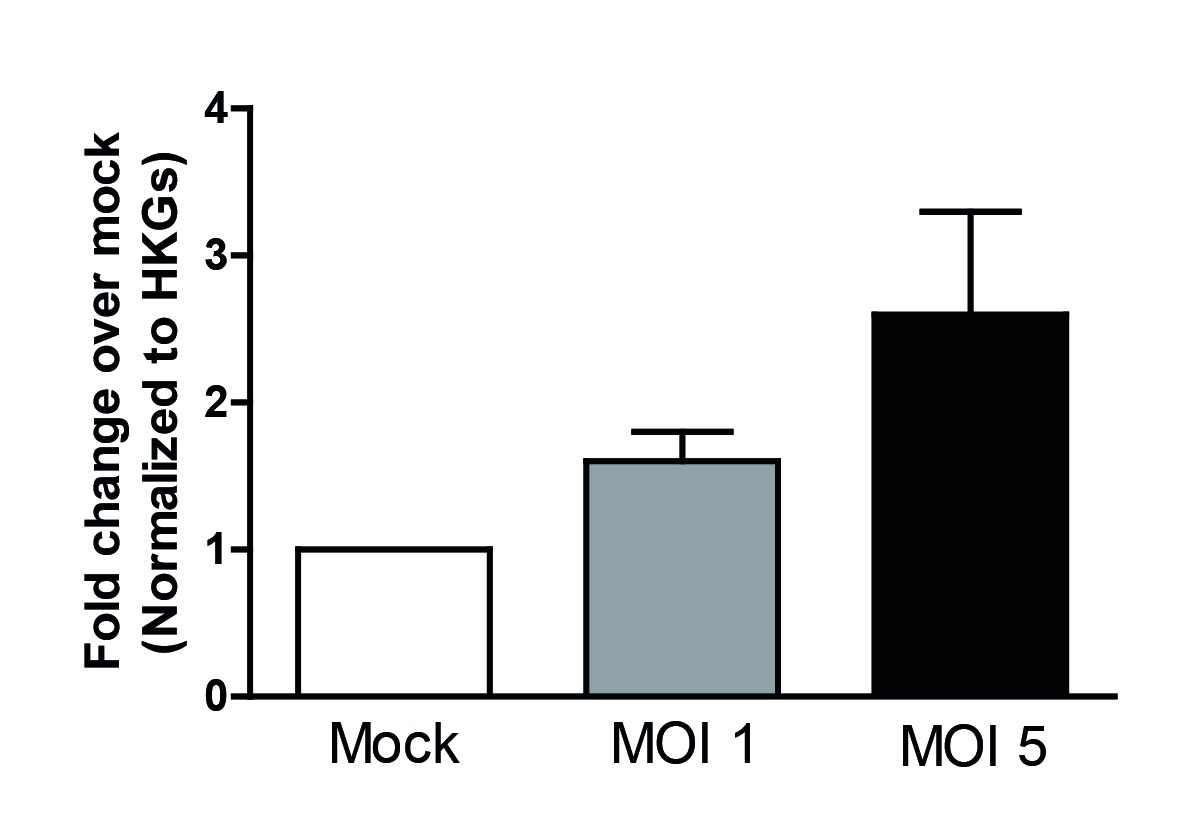

Supplement: S6 Fig — MDMs were infected with DENV at the indicated MOIs and at 24 hpi total RNA was extracted. Gene expression was investigated by microarray [33]. Probe values were normalized against the total signal intensity of the sample and subsequently, the fold change of the probes were expressed relative to the mock condition of the same donor taking into account the house keeping genes (HKGs) GAPDH, β-actin, β-glucuronidase, Hypoxanthine-guanine phosphoribosyltransferase and heat shock protein 90β1. Data shows mean ± SD from four different blood donors. (TIF) [file pntd.0005981.s006.tif]

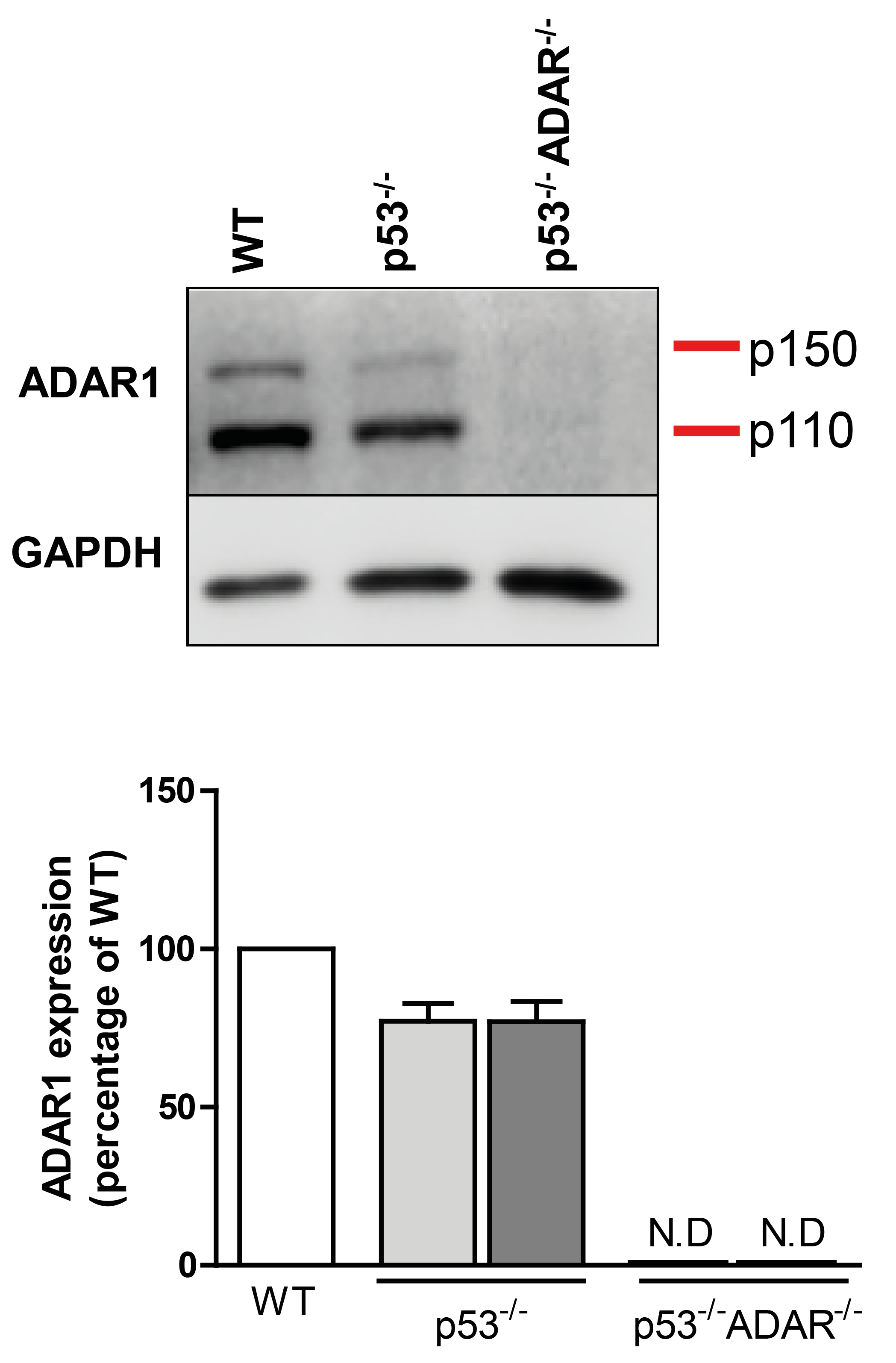

Supplement: S7 Fig — Representative blot of ADAR1 expression in cultured wild-type MEFs (WT), p53 KO MEFs (p53-/-) and p53/ADAR double KO MEFs (p53-/-ADAR-/-). The expression of ADAR1 was normalized to that of GAPDH and it is expressed as the percentage of the WT cells. Data shows mean ± SEM from three independent experiments. ND, no determined. (TIF) [file pntd.0005981.s007.tif]
